# Interfacial engineering of an amino-functionalized bimetallic NiNd-MOF decorated MoS_2_ heterostructure for enhanced symmetric supercapacitor performance

**DOI:** 10.1039/d6ra07975j

**Published:** 2026-10-15

**Authors:** Oviya Annamalai, Bhavani Sankaran, T. Kalaivani

**Affiliations:** a Department of Physics and Nanotechnology, College of Engineering and Technology, SRM Institute of Science and Technology Kattankulathur 603-203, Chengalpattu District Tamil Nadu India kalaivat@srmist.edu.in

## Abstract

Rational engineering of heterostructure electrode materials with optimized charge-transport pathways and abundant electroactive sites is highly desired for developing high-performance supercapacitor (SCs). In this work, a hierarchical NiNd-MOF@MoS_2_ hybrid architecture was successfully synthesised by a facile solvothermal strategy, exploiting the synergistic interfacial interactions between bimetallic NiNd-MOF and layered MoS_2_ nanosheets. The heterostructure construction not only inhibit the aggregation of nanosheets but also improve the active-site utilization and facilitate the ion/electron transfer efficiency in the electrode framework, which can accelerate the electrochemical reaction kinetics. Comprehensive structural, morphological, and compositional analyses confirmed the incorporation of MoS_2_ within the NiNd-MOF matrix and the formation of a stable hierarchical architecture. Benefiting from the synergistic integration of NiNd-MOF and MoS_2_, NiNd-MOF@MoS_2_ delivered a high specific capacitance (*C*_sp_) of 813 F g^−1^ at 1 A g^−1^, outperforming the pristine NiNd-MOF. Furthermore, the assembled symmetric two-electrode system exhibited a *C*_sp_ of 76.5 F g^−1^ at 1 A g^−1^ and energy density (*E*_d_) of 21 Wh kg^−1^ at a power density (*P*_d_) of 696.7 W kg^−1^ and it also demonstrated 100% coulombic efficiency and excellent cycling stability, retaining 86.9% of its initial capacitance after 10 000 charge–discharge cycles. Furthermore, the practical application of the NiNd-MOF@MoS_2_ hybrid was demonstrated in an LED with transistor assistance, further supporting its promising application for portable low power electronic device for next-generation energy-storage systems.

## Abstract 2

Rational engineering of heterostructure electrode materials with optimized charge-transport pathways and abundant electroactive sites is highly desired for developing high-performance supercapacitor (SCs).

## Graphical abstract

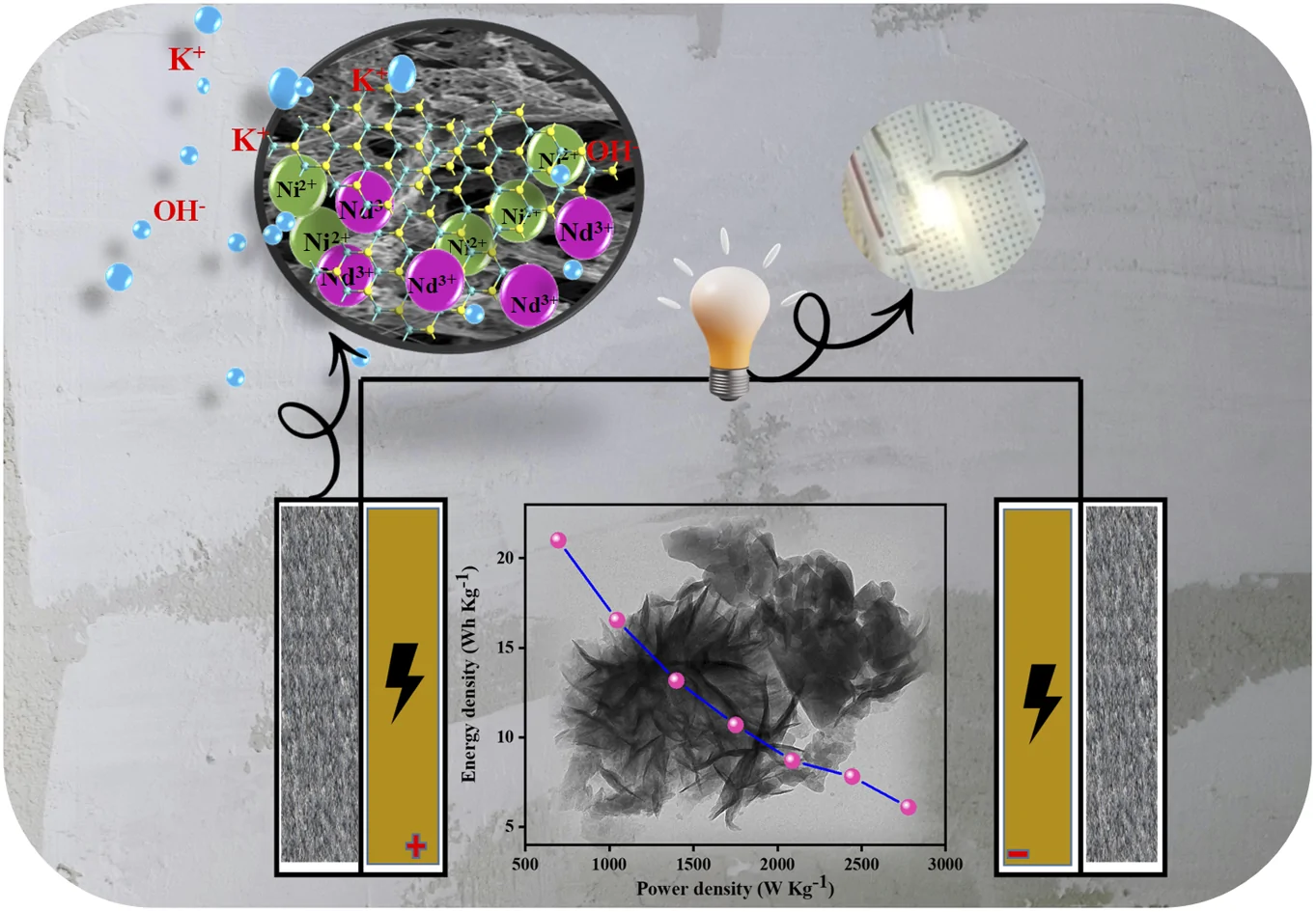

## 1. Introduction

Rapid exhaustion of fossil fuel reserves, coupled with the escalating environmental pollution and greenhouse gas emissions associated with their consumption has necessitated an urgent transition toward sustainable energy systems.^1–3^ In this respect, electrochemical energy storage and conversion systems like batteries, SCs, and fuel cells are considered more effective in order to proficiently address these problems.^4–6^ SCs are attractive among the various energy storage systems because they are capable of achieving high *P*_d_, extended life cycle, and fast charge–discharge capabilities.^7–9^ Therefore, the rational design of SCs and the improvement of electrode materials possessing high surface accessibility, excellent electron mobility, and optimized hierarchical porosity have become a focal point of contemporary research aimed at developing advanced energy storage systems.^10^ The past few years have witnessed two prominent categories of electrode materials being extensively researched for SCs applications.^11^ The first category consists of carbon substances like carbon nanotubes, carbon fibers, graphene, and porous carbon, in which charging occurs by non-faradaic electrostatic phenomena.^12–14^ The second category comprises redox-active compounds like transition metal oxides, hydroxides, and conductive polymers, here charging occurs by a reversible faradaic process.^15–17^ Carbon-based materials display prolonged cycling durability and elevated power output, however the *E*_d_ of these materials remain low owing to low capacitance in electric double layer capacitors.^18,19^ On the other hand, the redox-active compounds in pseudocapacitors make use of faradaic redox reactions, which are rapid and reversible thereby, the pseudocapacitive materials can provide higher *E*_d_ than EDLC. However, there was a problem concerning the materials due to the low cycling stability and rate capabilities.^20,21^ Consequently, to further enhance the performance of the SCs much more synthesis investigation is required in the fields of highly active nanostructured electrodes.^22^ Based on this, Metal Organic Frameworks (MOFs) have recently gained substantial attention as an innovative family of porous material achieved by the coordination of organic ligands with metal ions or metal clusters.^23–25^ They possess excellent ordered structures, tuneable pore size, and surface area. MOFs are not only utilized as materials for gas separation, catalysis, sensing, and drug delivery but also as electrodes for batteries and SC, so the redox properties of metal ions and the porous structure of these frameworks may be utilized.^26–28^ Several mono-metal MOFs with varied metal ions, such as the Ni, Co, Fe, Mn, and Cu, have been investigated as SCs electrodes.^29–31^ Although the capacitance values are promising, the relatively low intrinsic electrical conductivity and limited ion-diffusion kinetics of monometallic MOFs can hinder their electrochemical performance^32^ A variety of multi-metal or hybrid structures have been conceptualized until now in an attempt to address these limitations.^33^ Among transition metal-based MOFs, nickel-based MOFs have been widely investigated owing to the rich redox chemistry associated with the Ni^2+^/Ni^3+^couple.^34,35^ However, their application in electrochemical devices is limited by low electrical conductivity and structural degradation all through prolonged charge–discharge cycles.^36^ In contrast, rare earth-based MOFs have attracted significant interest due to unique chemistry of rare earth elements.^37^ Among them, Nd^3+^ is a promising metal center owing to its large ionic radius and high coordination number. However, their low intrinsic conductivity and limited redox activity associated to transition metal-based MOFs remain major drawbacks.^38^ Recently, bimetallic MOFs represents a rational strategy to overcome these limitations. As a result Nd^3+^ was deliberately selected as secondary metal, because of higher its charge, larger ionic size and different coordination features compared with Ni^2+^, giving a different metal-centre environment in the NH_2_-BDC framework. The flexible coordination behaviour of Nd^3+^ might affect the local metal–ligand configuration and structural features of the parent Ni-MOF, which provides a rational basis for the study of a Ni^2+^/Nd^3+^ bimetallic framework. The incorporation of Nd-MOF in Ni-MOF was considered to induce a synergistic effect, where the coexistence of Ni^2+^ and Nd^3+^ enhances the overall capacitance. The presence of both Ni^2+^ and Nd^3+^ in bimetallic MOF was recognized as effective in enhancing capacitance. Additionally, incorporation of 2D material such as MoS_2_, in the MOF structure could be a promising method in overcoming such limitations.^39,40^ MoS_2_ naturally has plenty of active sites for electrochemical reactions, high capacity, and strong chemical stability.^41^ The layered MoS_2_ nanosheets in MOFs offer the possibility of efficient electron transport pathways and help to maintain the structural stability for repeated charge–discharge processes.^42^

Recent developments have focused on electrochemical benefits offered by transition metal based MOFs, rare-earth MOFs, and MoS_2_ composites as highly potential SCs materials. In particular, Wang *et al.*, found that a Ni-MOF provided a *C*_s_ of around 402 F g^−1^, showing its pseudocapacitive nature; however, its efficiency could be hindered due to low electrical conductivity.^43^ On the other hand, rare-earth metal based MOFs have received considerable interest recently because of their electronic structure and various oxidation levels that increase the redox activity. For instance, Singh *et al.* have shown that a rare earth MOF of Eu-based H_2_L has a capacitance of 560 F g^−1^ at 1 A g^−1^.^44^ Huang *et al.*, noted that the hydrothermal technique was employed in the preparation of MoS_2_ nanosheets whose electrochemical properties were investigated regarding the value of *C*_s_ of 129.2 F g^−1^ at 1 A g^−1^. Moreover, its electrochemical behaviour is attributed to its high surface area and unique 2D structure.^45^ However, Ni-MOF@MoS_2_ and other MOF@MoS_2_ heterostructures have already been investigated for SC application, indicating that the mere combination of MoS_2_ with a Ni based MOF is not, by itself, sufficient to establish distinct scientific novelty. In the present strategy, the Ni^2+^/Nd^3+^ bimetallic framework is integrated with layered MoS_2_ to construct a chemically differentiated heterostructure and to potentially improve the electrochemical charge storage and charge-transfer behaviour. Therefore, the novelty is not only the incorporation of MoS_2_ but also the use of a Ni^2+^/Nd^3+^ bimetallic NH_2_-BDC framework as MOF component. Ni-MOF, Nd-MOF, NiNd-MOF, MoS_2_, and NiNd-MOF@MoS_2_ are systematically compared, so as to separately evaluate the contributions from the Nd incorporation and the subsequent MoS_2_ integration.

In this study, novel NiNd-MOF@MoS_2_ heterostructure was synthesized by solvothermal approach to integrate Ni^2+^/Nd^3+^ bimetallic coordination chemistry with the layered structure of MoS_2_. This architecture was designed to understand the effect of the Ni^2+^/Nd^3+^ bimetallic framework and the formed heterostructure on charge transfer, ion transport, and electrochemical stability, rather than directly mixing a Ni based MOF with MoS_2_. The structure–property relationship was systematically investigated through structural, morphological, surface-chemical and electrochemical analyses. As a result, NiNd-MOF@MoS_2_ delivers a *C*_sp_ of 813 F g^−1^ in the three-electrode configuration. In addition, the symmetric two-electrode system assembled with NiNd-MOF@MoS_2_ exhibits a *C*_sp_ of 76.5 F g^−1^ at 1 A g^−1^ with an *E*_d_ of 21 Wh kg^−1^ with a corresponding *P*_d_ of 696.7 W kg^−1^. Moreover, the device shows 86.95% capacitance retention and 100% coulombic efficiency over 10 000 charge–discharge cycles. The assembled two-electrode configuration also shows a stable voltage output under the LED illumination, indicating its potential for practical application in energy storage and electronic devices.

## 2. Methodological section

### 2.1. Synthesis of NiNd-MOFMoS_2_ composite

NiNd-MOF@MoS_2_ composite was synthesized by one-pot solvothermal assembly using pre-synthesized MoS_2_ technique. 0.5 g of Ni (NO_3_)_2_·6H_2_O and 0.5 g of Nd (NO_3_)_3_·6H_2_O were blended with 50 mL of DMF and ethanol (1 : 1). It was stirred constantly until it transformed into a uniform solution and then MoS_2_ was introduced to form a mixed solution. The gained homogeneous suspension was sealed in a hydrothermal reactor at 140 °C −24 h followed by washing with ethanol and DI. The NiNd-MOF@MoS_2_ composite was then left to dry overnight at 70 °C (Fig. 1).

**Fig. 1:**
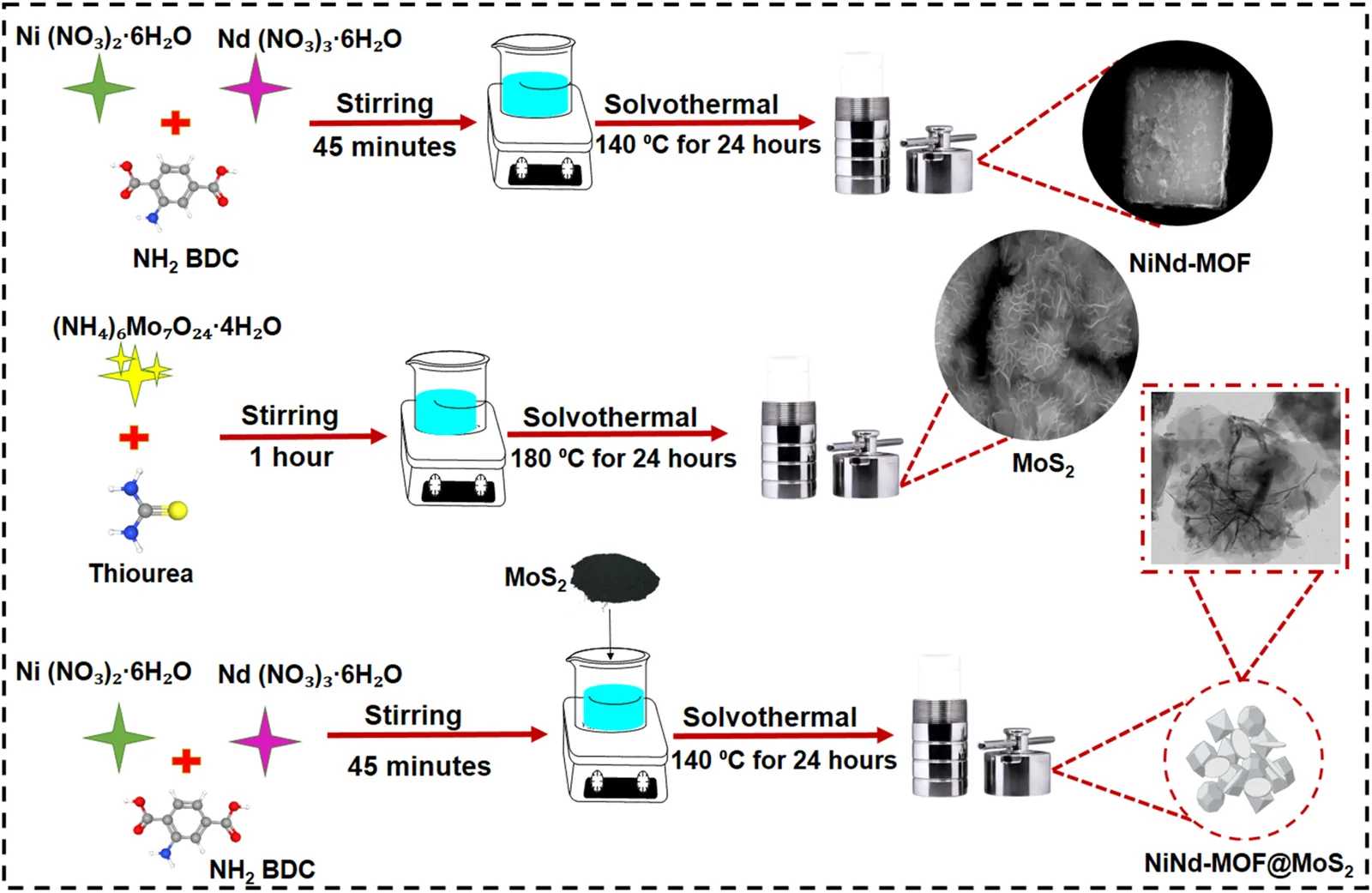
Graphical diagram for the preparation of NiNd-MOF, MoS_2_, NiNd-MOF@MoS_2_.

Comprehensive details on the chemicals, synthesis procedure for NiNd-MOF, MoS_2_, electrochemical measurements, and electrode assembly, characterization techniques are described in the supporting information (SI).

## 3. Results and discussion

### 3.1. Physical and chemical investigation

Phase purity and crystallite size of the synthesised Ni-MOF, Nd-MOF, NiNd-MOF, MoS_2_, and NiNd-MOF@MoS_2_ were analysed by X-ray diffraction. Ni-MOF shows diffraction peaks at 2*θ* = 10.7°, 16.2°, 19.4°, 22.5°, 25.7°, 31.0°, 34.7°, 42.4°, 53.1° corresponding to the planes (211), (002), (001), (600), (004), (100), (022), (512), and (102) respectively, in agreement with the reported Ni-NH_2_-BDC MOF diffraction data, confirming the crystalline nature of Ni-MOF (JCPDS card no. 02-1216).^46^ Nd-MOF shows the diffraction peaks at 2*θ* = 9.6°, 12.2°, 19.6°, 21.5° 24.1°, and 26.1° corresponding to (011), (111), (112), (220), (113) and (131) planes respectively, in accordance with the reported {[Nd_2_ (NH_2_-BDC)_3_ (DMF)_4_]}_*n*_ (CCDC card no. 1498826), which exhibits triclinic crystal structure.^47^ NiNd-MOF shows the characteristic peaks of the crystalline structure of the bimetallic network, consistent with a crystalline Ni^2+^/Nd^3+^ containing NH_2_-BDC coordination framework. MoS_2_ peaks at 2*θ* = 13.2°, 23.5°, 31.7°, and 56.8° indicate the (002), (101), (103), and (110) planes respectively consistent with the layered MoS_2_ hexagonal structure (JCPDS card no. 37-1492).^48^ XRD pattern of NiNd-MOF@MoS_2_, confirms the typical diffraction peaks of all the individual materials, indicating the coexistence of the characteristic crystalline features of NiNd-MOF and MoS_2_ in the composite (Fig. 2(a)). Therefore, the structural evolution can be attributed as the integration of the Ni^2+/^Nd^3+^ containing NH_2_-BDC coordination framework with the layered MoS_2_ phase. Crystallite sizes of Ni-MOF, Nd-MOF, NiNd-MOF, MoS_2_, and NiNd-MOF@MoS_2_ were calculated using the Scherer equation:

1 *D* = 0.9*λ*/*β* cos *θ*

where *K* = 0.9, *λ* = 0.1540 nm, *β* = FWHM, and *θ* = Bragg diffraction angle.^49^ The estimated domain sizes of Ni-MOF, Nd-MOF, NiNd-MOF, MoS_2_ and NiNd-MOF@MoS_2_ are 6.5, 17.1, 21.3, 3.2 and 34.5 nm respectively. Considerably greater crystallite size of NiNd-MOF@MoS_2_ relative to the individual components indicates a higher degree of ordering in the structure after the formation of the heterostructure. Improved crystallinity is likely to facilitate effective charge-transport pathway and improved structural robustness over successive charge–discharge cycles.^50^ However, the small size of MoS_2_ crystallites leads to a high density of exposed edge-active sites and short ion-diffusion pathways, advantageous for electrochemical reactions. Therefore, the combination of MoS_2_ nanosheets and bimetallic NiNd-MOF framework can integrate the abundance of electroactive sites and the enhanced structural stability. The synergistic architecture is expected to promote the efficient ion/electron transport and fast charge storage kinetics, resulting in the improved electrochemical performance of the NiNd-MOF@MoS_2_ electrode.

**Fig. 2:**
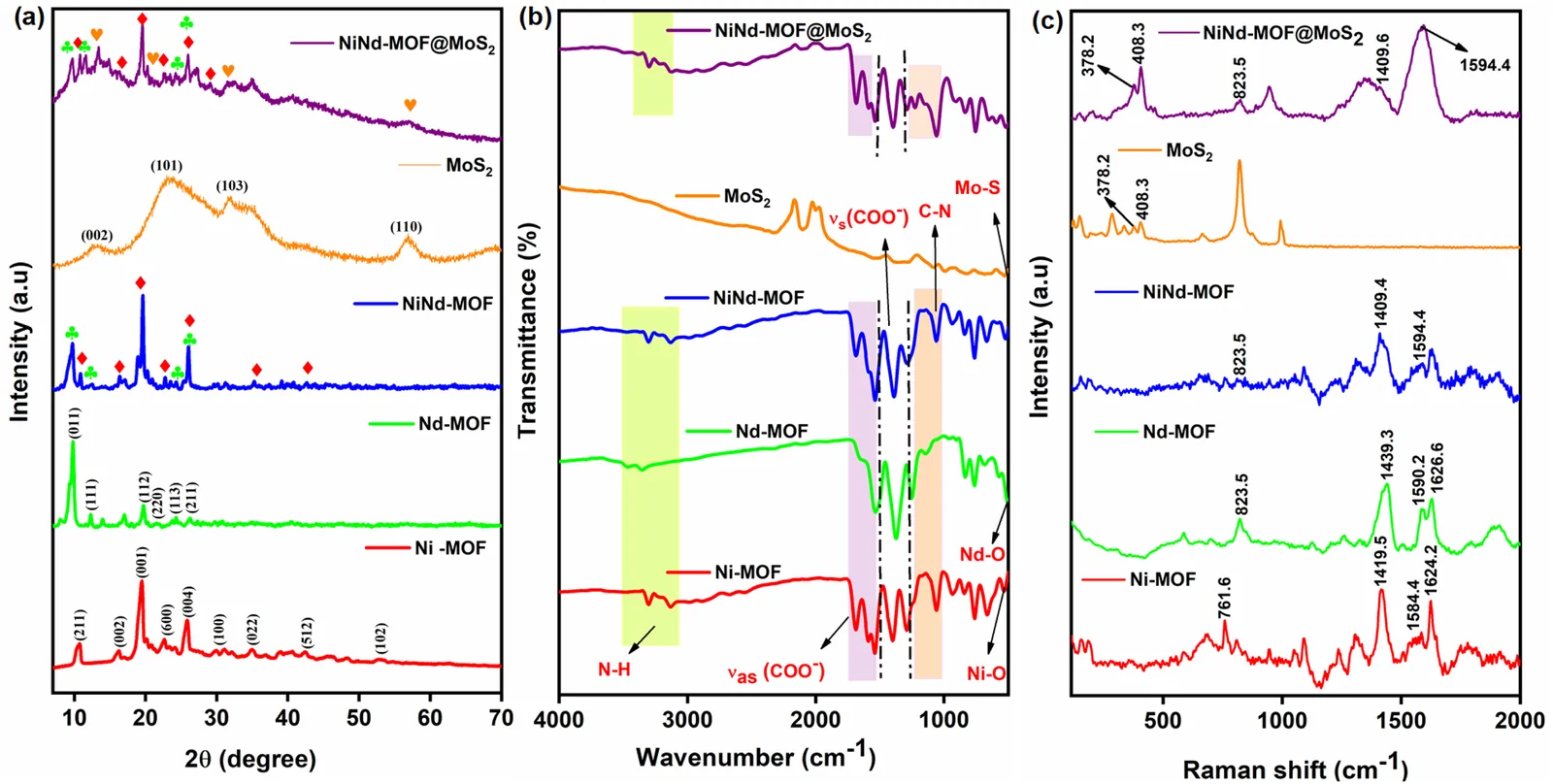
(a) XRD patterns, (b) FT-IR, (c) Raman spectra of Ni-MOF, Nd-MOF, NiNd-MOF, MoS_2_ and NiNd-MOF@MoS_2_.

Fourier-transform infrared (FT-IR) was employed to investigate the functional groups and coordination environment of the synthesized materials. A broad adsorption band in the 3000–3500 cm^−1^ region, is attributed to the N–H stretching vibration of the amino groups of NH_2_-BDC linker confirming the retention of amino functionality in all MOF based samples.^51^ The carboxylate groups (–COO^−^) showed both the asymmetric and symmetric stretching vibrations, *ν*_as_(COO^−^) being at 1620–1550 cm^−1^ (Ni-MOF),^52^ 1615–1540 cm^−1^ (Nd-MOF),^53^ and around 1598 cm^−1^ (NiNd-MOF), while *ν*_s_(COO^−^) bands were positioned in the range of 1425–1300 cm^−1^. The co-presence of the two metal ions (Ni^2+^ and Nd^3+^) induces small shifts of the carboxylate stretching vibrations, pointing to the changes of the local coordination environment due to the incorporation of both metal ions in the framework. 1250–1000 cm^−1^ absorptions were assigned to aromatic skeleton vibrations and C–N stretching of amino groups, hence confirming the existence of NH_2_-BDC. Low-frequency region exhibited intense Ni–O and Nd–O vibrations (500–600 cm^−1^) indicating intense metal–ligand interaction within the frameworks, while MoS_2_ exhibits Mo–S stretching vibrations at 500–450 cm^−1^.^54^ The FT-IR spectrum of NiNd-MOF@MoS_2_ reveals the characteristic vibrational modes of NiNd-MOF and MoS_2_, confirming the successful formation of NiNd-MOF@MoS_2_ without disturbing the structural integrity of the pristine materials. Furthermore, the changes of band position and the intensity are small, indicating the interfacial interactions between the amino-functionalized NiNd-MOF framework and the MoS_2_ nanosheets. All these results proved the targeted formation of NiNd-MOF@MoS_2_ heterostructure and its potential application as electrode material for SCs (Fig. 2(b)).

The vibrational characteristics of the synthesized materials were also studied by Raman spectroscopy, with particular emphasis on the structural features of MoS_2_ before and after composite formation. The Raman bands of the linkers are characteristic of Ni-MOF at 761.6, 1419.5, 1584.4, and 1624.2 cm^−1^, Nd-MOF at 823.5, 1439.3, 1590.2, and 1626.6 cm^−1^, and NiNd-MOF at 823.5, 1409.4, and 1594.4 cm^−1^.^55^ Pristine MoS_2_ displays the characteristic E_2g_^1^ and A_1g_ modes of MoS_2_ at 378.2 and 408.3 cm^−1^ respectively.^56^ Notably, the two MoS_2_ modes are preserved in NiNd-MOF@MoS_2_, indicating the preservation of the characteristic vibrational features of the MoS_2_ phase after composite formation. In addition, the composite exhibits the characteristic bands of NiNd-MOF. Therefore the characteristic Raman signatures of MoS_2_ and NiNd-MOF are simultaneously preserved, providing complementary evidence for the incorporation of both components in the NiNd-MOF@MoS_2_ (Fig. 2(c)).

### 3.2. Morphological and surface area analysis

Ni-MOF, Nd-MOF, NiNd-MOF, MoS_2_, and NiNd-MOF@MoS_2_ were examined in detail for the morphological features using HR-SEM and is presented in Fig. 3. Ni-MOF presents nanosheet-like morphology, where ultrathin sheets form a network. This can lead to a large exposed surface area and multiple electro active sites (Fig. 3(a) and (b)). In contrast, Nd-MOF exhibits regular cubic morphology with a smooth surface and sharp edges, indicating the formation of a highly ordered crystalline framework (Fig. 3(c) and (d)). Upon incorporation of both the Ni^2+^ and Nd^3+^ ions the resulting NiNd-MOF preserves its cubic structure with a rough surface and some protrusions such as nanosheets suggesting the successful synthesis of a bimetallic MOF with improved surface roughness (Fig. 3(e) and (f)). MoS_2_ exhibited a clear hierarchical flower-like structure with ultrathin, crumpled nanosheets.^57^ Layered architectures can offer abundant surface active sites and allow for electrolyte penetration, thereby facilitating electrochemical charge storage processes (Fig. 3(g) and (h)).^58^ NiNd-MOF@MoS_2_ composite presents a hierarchical heterostructure of tightly-coupled NiNd-MOF cubes and MoS_2_ nanosheets (Fig. 3(i) and (j)). The close contact of these components at the interface indicates successful hybridization and porous and interconnected architecture. The unique morphology is predicted to provide abundant electrochemically active sites, facilitate diffusion of electrolyte, and allow efficient ion/electron transport during charge–discharge processes. In addition, the EDX spectrum and elemental mapping corroborates the presence of Ni, Nd, Mo, S, C, O and N, which further supports the formation of NiNd-MOF@MoS_2_ (Fig. 3(i)–(viii)).

**Fig. 3:**
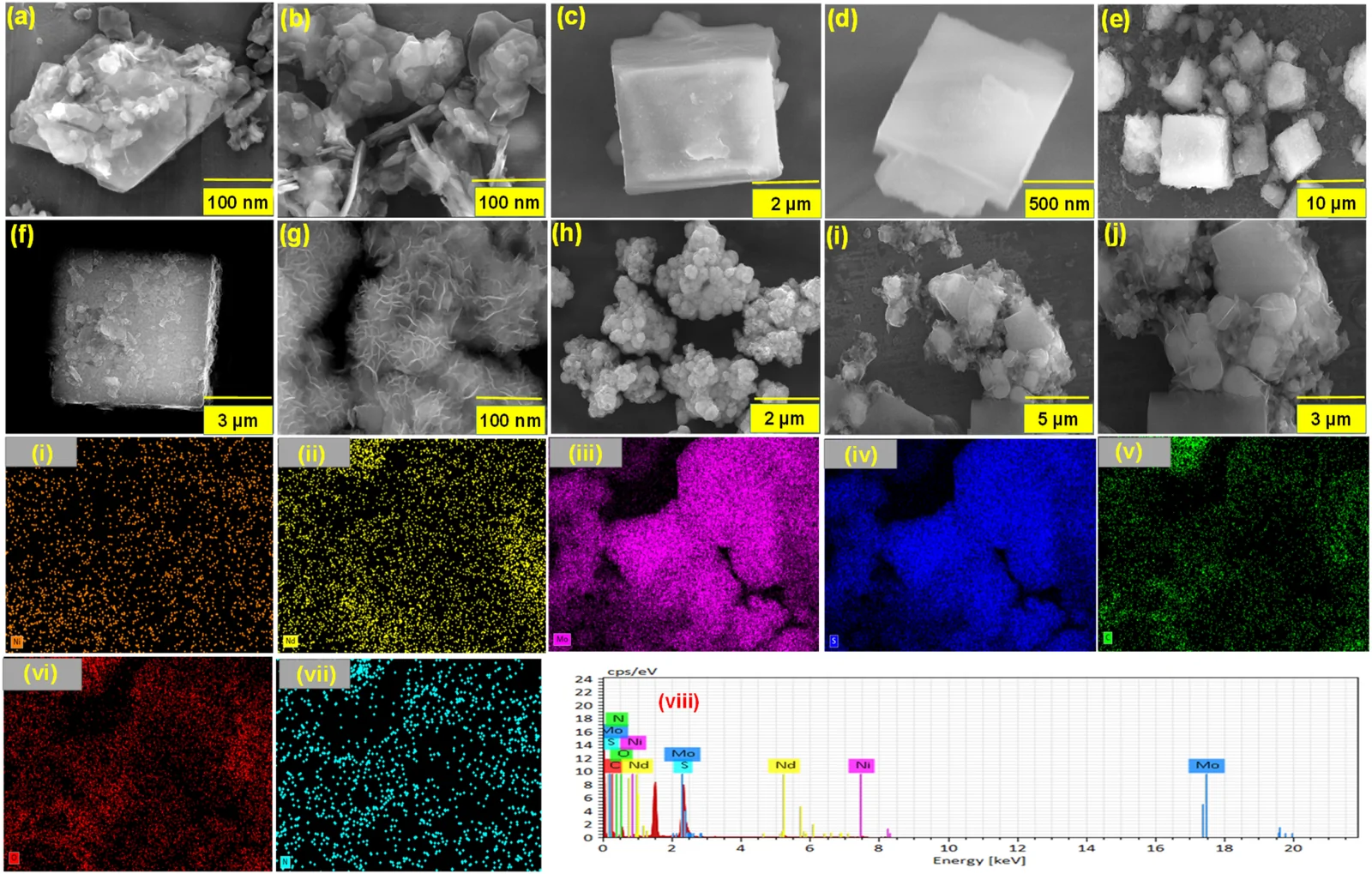
HR-SEM images of (a) and (b) Ni-MOF, (c) and (d) Nd-MOF, (e) and (f) NiNd-MOF, (g) and (h) MoS_2_, (i) and (j) NiNd-MOF@MoS_2_, (i)–(vii) EDX elemental mapping, and (viii) EDX spectrum.

To further elucidate the morphological properties of Ni-MOF, Nd-MOF, NiNd-MOF, MoS_2_ and NiNd-MOF@MoS_2_ HR-TEM, EDX and SAED analysis were performed and it is presented in Fig. 4(a)–(q). Ni-MOF has a sheet-like morphology which is characteristic of crystalline MOF structures (Fig. 4(a) and (b)). Whereas the Nd-MOF consists of agglomerated irregular shaped nanoparticles (Fig. 4(c) and (d)). The bimetallic NiNd-MOF (Fig. 4(e) and (f)) exhibits a similar morphology to the monometallic counterparts, indicating that Nd incorporation does not induce significant changes in the framework structure. The MoS_2_ images show thin, wrinkled and transparent nanosheets indicating few-layer architectures with high surface area (Fig. 4(g) and (h)). The NiNd-MOF particles are uniformly distributed on the MoS_2_ nanosheets in the NiNd-MOF@MoS_2_ (Fig. 4(i) and (j)), indicating the close contact of NiNd-MOF with MoS_2_ nanosheets and formation of the heterostructure. The presence of crystallinity and the coexistence of the constituent phases was further confirmed by HR-TEM. The lattice fringes of pristine MoS_2_ are 0.615, 0.226 and 0.180 nm corresponding to the (002), (103) and (110) planes, respectively.^59^ NiNd-MOF shows lattice spacing of 0.266 nm and 0.361 nm, which correspond to (022) and (004) planes. In NiNd-MOF@MoS_2_, the typical 0.615 nm (002) fringe of MoS_2_ and 0.266 nm (022) and 0.361 nm (004) fringes of the NiNd-MOF are preserved, which confirms the co-existence (Fig. 4(k)–(m)). In the SAED pattern, no pronounced bright diffraction rings were observed, for NiNd-MOF, but broad diffuse features, assignable to the high electron-beam sensitivity of MOF frameworks and the resultant beam-induced structural degradation during TEM acquisition.^60,61^ MoS_2_ exhibits concentric diffraction rings, where the measured inner ring corresponds to ∼0.16 nm, which is indexed to the (110) plane in agreement with the reported diffraction data of MoS_2_.^62^ The appearance of the MoS_2_ diffraction peaks in NiNd-MOF@MoS_2_ suggests that the MoS_2_ crystalline phase is retained after hybridization (Fig. 4(n)–(p)). Further EDX spectrum also confirmed the presence of Ni, Nd, Mo, S, C, N and O (Fig. 4(q)).

**Fig. 4:**
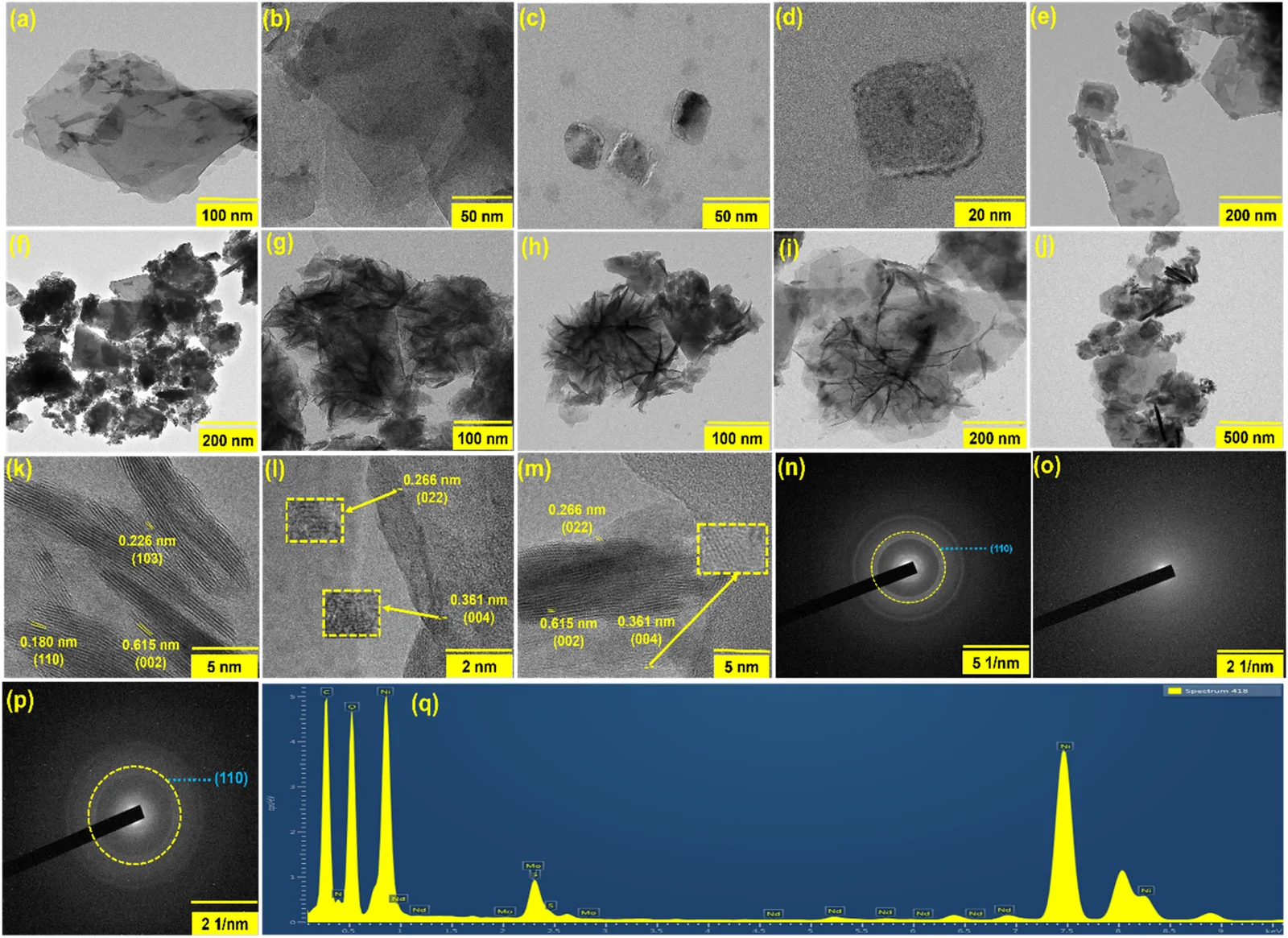
HR-TEM images of (a) & (b) Ni-MOF, (c) & (d) Nd-MOF, (e) & (f) NiNd-MOF, (g) & (h) MoS_2_, (i) & (j) NiNd-MOF@MoS_2_, (k)–(m) lattice images of MoS_2_, NiNd-MOF, NiNd-MOF@MoS_2_. (n)–(p) SAED patterns of NiNd-MOF, MoS_2_, NiNd-MOF@MoS_2_, (q) EDX spectrum.

The surface chemical composition and electronic states of NiNd-MOF@MoS_2_ were systematically characterized by XPS. The survey spectrum indicates the successful formation of NiNd-MOF@MoS_2_ without any impurity peaks, which means the Ni, Nd, Mo, S, C, N and O elements are successfully formed (Fig. 5(a)). XPS spectrum of the Ni 2p region showed two spin–orbit peaks for Ni 2p_3/2_ and Ni 2p_1/2_ with binding energies of 855.7 eV and 873.3 eV respectively. The observed spin–orbit splitting of Δ*E* = 17.6 eV and the characteristic satellite peaks at 861.0 and 879.4 eV are consistent with Ni^2+^ species coordinated in the MOF framework (Fig. 5(b)).^63^Fig. 5(c) shows the deconvoluted Nd 3d spectrum which displays two intense peaks at 982.8 eV and 1005.6 eV corresponding to Nd 3d_5/2_ and Nd 3d_3/2_, respectively. The spin–orbit splitting energy of Δ*E* = 22.8 eV is typical for Nd^3+^ species, indicating appropriate incorporation of Nd ions within the NiNd-MOF framework.^64^ The high resolution Mo 3d spectrum (Fig. 5(d)) displays two typical peaks at 228.6 and 232.6 eV, which are assigned to Mo^4+^ 3d_5/2_ and Mo^4+^ 3d_3/2_, respectively, indicating the existence of MoS_2_ in the heterostructure. Furthermore, a weak feature at ∼235.5 eV is assigned to a small amount of Mo^6+^ species, which has often been attributed to small surface oxidation of MoS_2_ after air exposure. The Mo^4+^ state dominance indicates the successful incorporation of the common chemical environment of the MoS_2_ in the NiNd-MOF@MoS_2_ and its retention.^65^ Two characteristic peaks at 161.6 eV and 162.8 eV attributed to S 2p_3/2_ and S 2p_1/2_ in the S 2p region shown in Fig. 5(e) are respective to S^2−^ species from MoS_2_.^66^ The anticipated spin–orbit interaction of sulfur in sulfide conditions is validated by the Δ*E* of 1.2 eV. Confirmation for the existence of residual organic linkers and carbonaceous fragments of the MOF backbone is provided by the three deconvoluted peaks in the C 1s spectra in Fig. 5(f) at binding energies of 284.6 eV, 285.8 eV, and 288.4 eV agreeing to C–C/C=C, C–O, and C=O bonds, respectively.^67^ Two O 1s peaks at 531.4 eV and 532.7 eV in Fig. 5(g) are due to the metal–oxygen (M–O) and C–O/C=O species transitions, which confirms the oxygen-containing functional groups and the metal–oxygen coordination in the composite.^68^ The highly intense high-energy peak at 399.6 eV in the N 1s core-level spectrum in Fig. 5(h) is attributed to coordinated nitrogen species of the NH_2_ functional group of the 2-aminoterephthalic acid linker, hence explaining evidence of successful insertion of the amino functionalized linker into the MOF framework.^69^

**Fig. 5:**
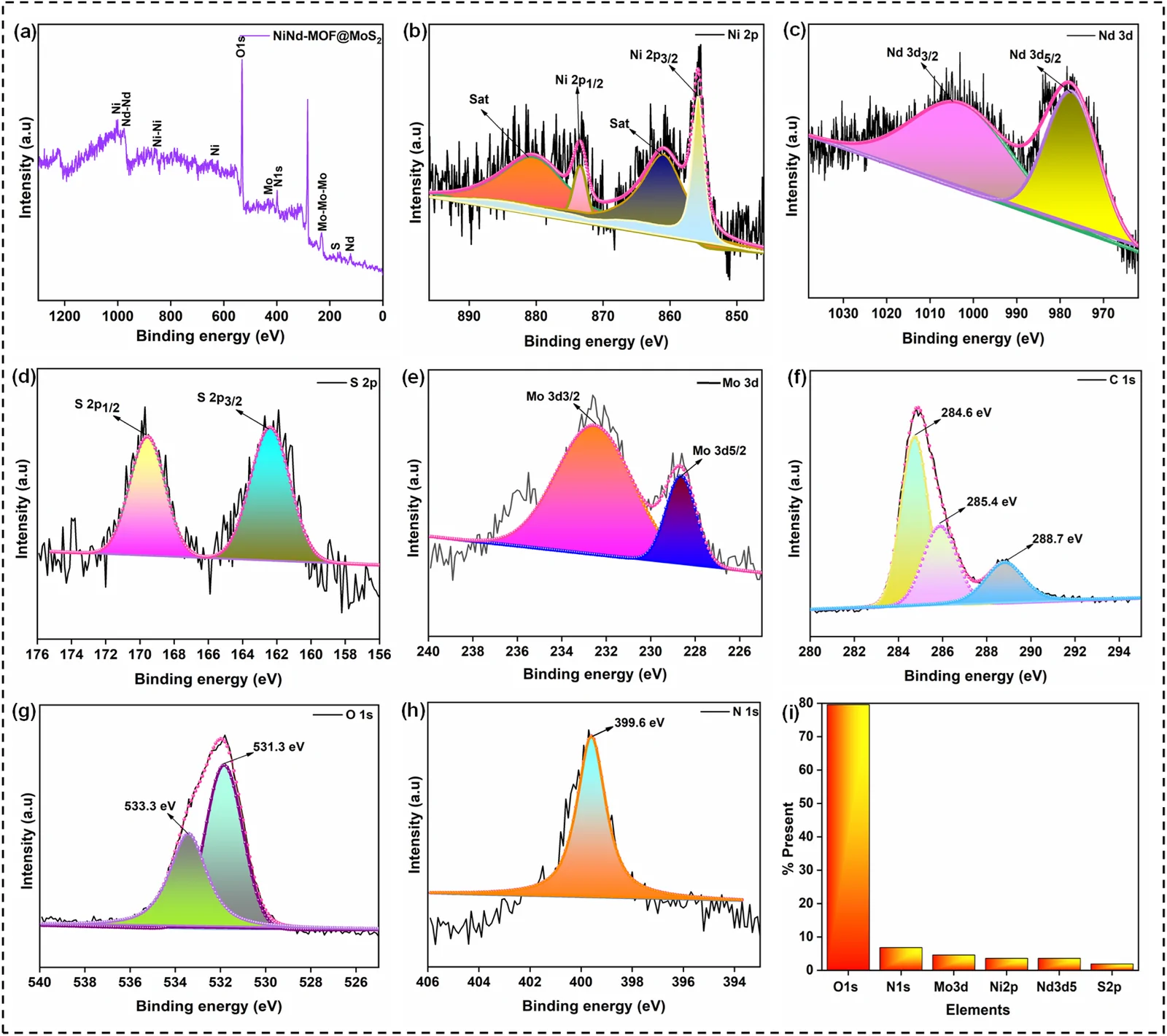
XPS profiles of NiNd-MOF@MoS_2_ (a) overall XPS survey spectrum, (b) Ni 2p, (c) Nd 3d, (d) S 2p, (e) Mo 3d, (f) C 1s, (g) O 1s, (h) N 1s, (i) elemental percentage.

Mesoporous properties of NiNd-MOF@MoS_2_ were studied by N_2_ adsorption–desorption isotherm based on Brunauer–Emmett–Teller (BET) method (Fig. 6(a)). The adsorption–desorption isotherm is of type IV according to the IUPAC classification, which suggests the mesoporous feature. The NiNd-MOF@MoS_2_ composite has a specific surface area of 100.5 m^2^ g^−1^, which offers many electrochemically accessible sites for the interaction with the electrolyte. The corresponding BJH pore size distribution curve (Fig. 6(b)) is used to verify the mesoporous nature of the heterostructure with an average pore radius of 13.845 Å and a pore volume of 0.382 cm^3^ g^−1^. This kind of hierarchical porous architecture facilitate electrolyte infiltration and shorten ion-transport pathways, which can favour the efficient ion diffusion and be conducive to the enhanced electrochemical performance of NiNd-MOF@MoS_2_ electrode.

**Fig. 6:**
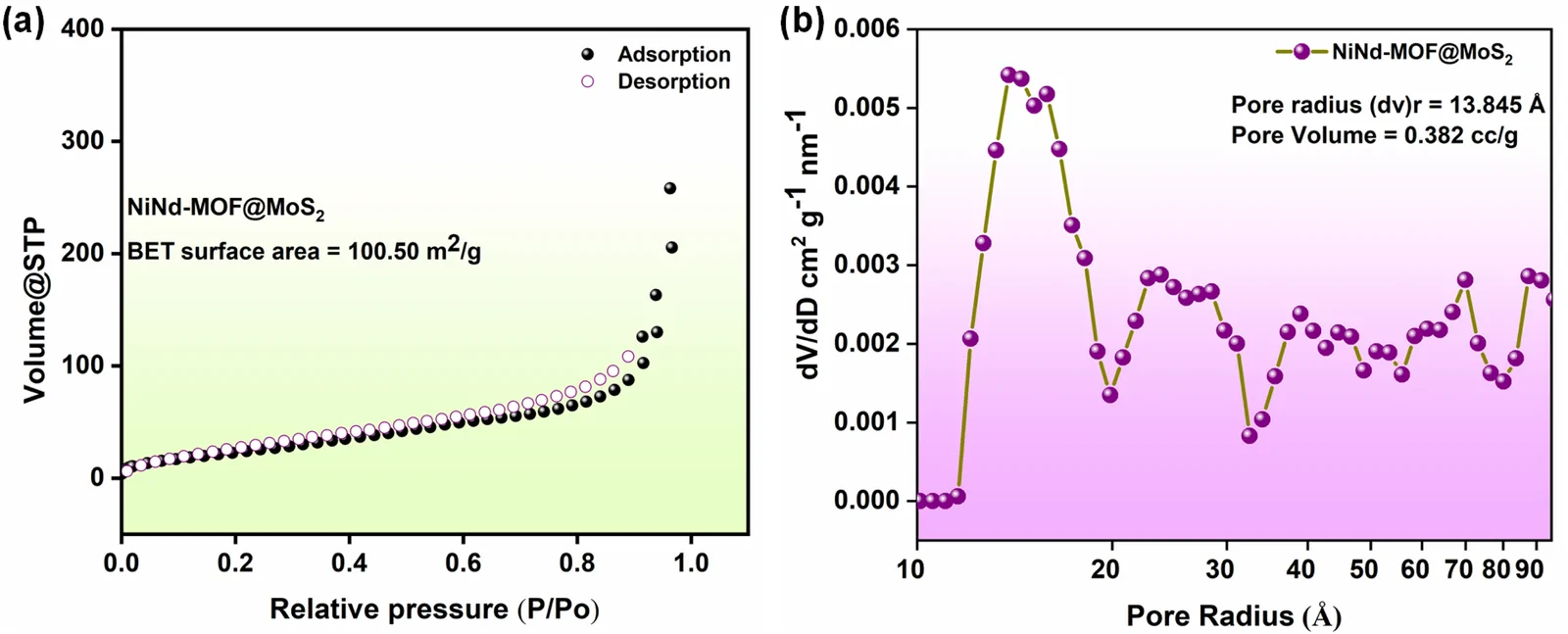
(a) BET analysis of N_2_ adsorption–desorption isotherm of NiNd-MOF@MoS_2_, (b) pore size distribution.

### 3.3. Electrochemical analysis

#### 3.3.1. Three-electrode studies

The electrochemical charge-storage performance of all electrodes was evaluated in three electrode configurations in 3 M KOH aqueous electrolyte with an optimized mass loading of ∼1.7 mg cm^−2^. In instances, cyclic voltammetry (CV) were done for Ni-MOF, Nd-MOF (Fig. S2(a) & (b)), NiNd-MOF, MoS_2_ and NiNd-MOF@MoS_2_ (Fig. 7(a–c)) at scan rates of 5–100 mV s^−1^ with the V *vs.* Ag/AgCl as a reference electrode. All electrodes exhibit distinct redox features, indicating a significant contribution of faradaic pseudocapacitive processes to charge storage. The redox behaviour of Ni-MOF is mainly derived from the reversible Ni^2+^/Ni^3+^ conversion, which provides rich faradaic charge-storage sites. As the scan rate increases, the current response increases gradually with a slight shift of the redox peaks, indicating the rapid reaction kinetics and good electrochemical reversibility.^70^ Nd-MOF exhibits relatively low current response and smaller enclosed CV area, suggesting limited electrochemical utilization of active sites. MoS_2_ displays broad anodic and cathodic redox features, which specifies considerable faradaic charge storage. Layered structure provides accessible surface and edge sites, which enhance electrolyte ion accessibility and charge storage.^71^ Incorporation of Nd^3+^ into Ni results in the increased CV area and current response in comparison with the pristine MOFs, which reveals the increased use of electroactive sites Ni. NiNd-MOF@MoS_2_ exhibits a larger CV area and higher peak current, indicating the improved charge-storage activity. The redox features are well maintained and the current response increases with scan rate, further suggesting good electrochemical kinetics and reversible faradaic charge storage. Faradaic reaction of Ni-based electrodes in 3 M KOH is described in eqn (2),^72^ while the redox contribution of Nd-MOF and MoS_2_ is discussed without marking the oxidation state transitions.

2 Ni(OH)_2_ + OH ↔ NiOOH + H_2_O + e^−^

**Fig. 7:**
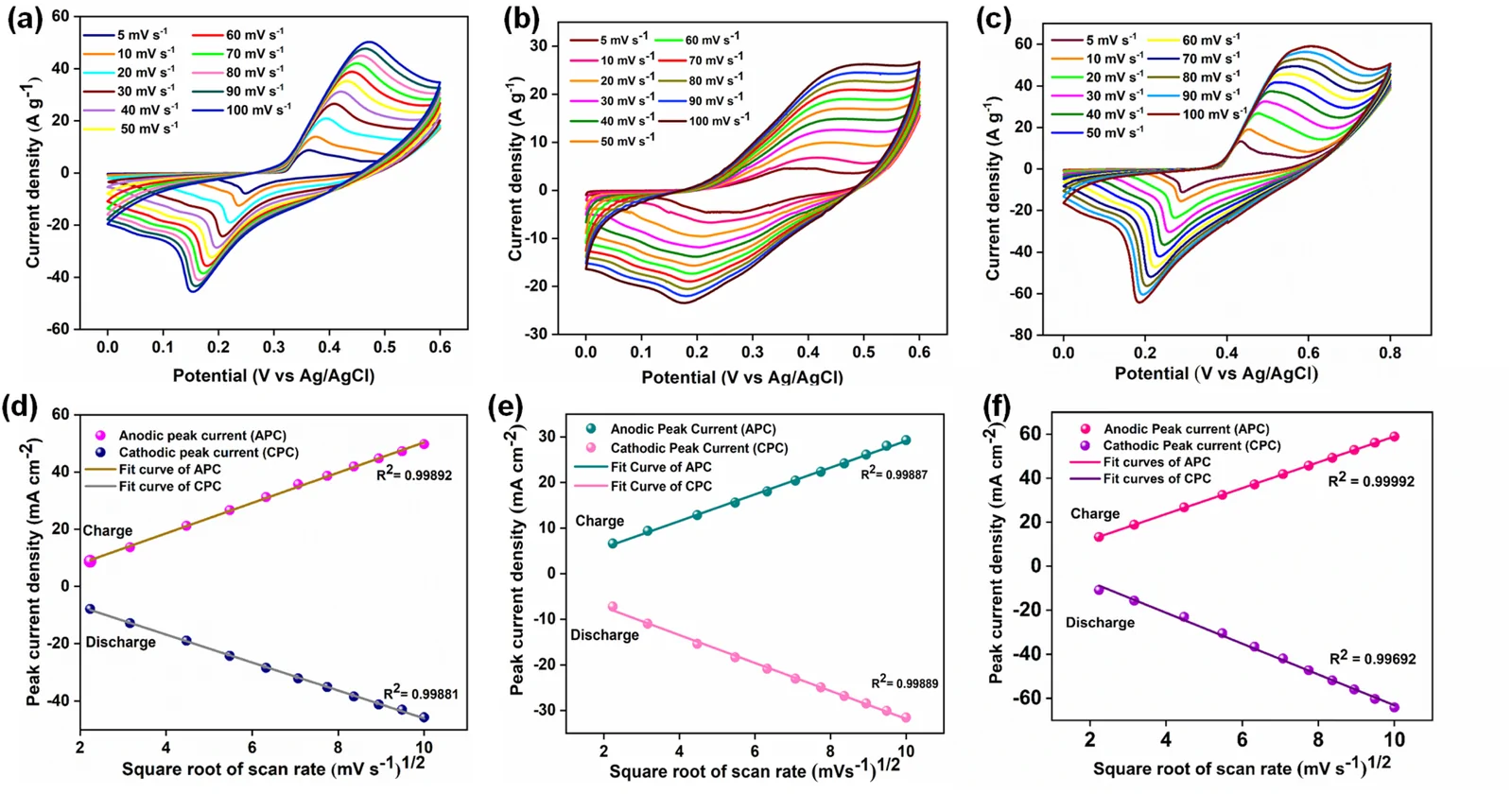
(a)–(c) CV of NiNd-MOF, MoS_2_, NiNd-MOF@MoS_2_ at 5–100 mV s^−1^, (d)–(f) *i*_p_*vs. v*^1/2^.

To further elucidate the kinetics of the faradaic charge-storage processes Randles–Sevcik type analysis was employed by correlating the anodic and cathodic peak currents (*i*_p_) with the square root of scan rate (*v*^1/2^).^73^ All electrodes show (Fig. S2(c), (d) and 7(d–f)), strong linear relationships for both oxidation and reduction processes, suggesting a significant diffusion-related contribution to the observed faradaic response.

The *b*-value was obtained from the power-law relationship,

3 *i*_p_ = *av*^*b*^

where *i*_p_ is peak current, and *v* is scan rate and *b* is a measure of the relative contribution of diffusion-related and surface-controlled processes. The *b* values were determined from the slopes of the log(*i*_p_) *vs.* log(*ν*) plots. Ni-MOF exhibits *b* = 0.616 indicating mixed charge-storage kinetics with a substantial diffusion related contribution, Nd-MOF shows *b* = 0.896 indicating major surface-controlled response (Fig. S3(a) & (d)). NiNd-MOF exhibits a larger diffusion contribution with *b* = 0.567 and MoS_2_ displays a similar diffusion contribution with a *b*-value of 0.578. Notably, NiNd-MOF@MoS_2_ exhibits *b* = 0.549, indicating the maximum diffusion related character among all studied electrodes (Fig. 8(a)–(c)). To further quantify the surface-controlled and diffusion-related charge-storage contributions, Dunn's analysis was performed using.

4 *i*(*V*) = *k*_1_*v* + *k*_2_*v*^1/2^

where *k*_1_*v* and *k*_2_*v*^1/2^ are the corresponding current contributions. The surface-controlled contribution increases with scan rate from ∼10–33% for Ni-MOF, ∼14–42% for Nd-MOF (Fig. S3(c) & (f)), ∼7–26% for NiNd-MOF, and ∼13–41% for MoS_2_. Conversely, NiNd-MOF@MoS_2_ displays the lowest surface-controlled contribution (∼6–20%), and maintains the largest diffusion-related contribution (Fig. 8(d)–(i)). This lower surface-controlled fraction is consistent with predominantly diffusion-associated charge-storage behaviour.

**Fig. 8:**
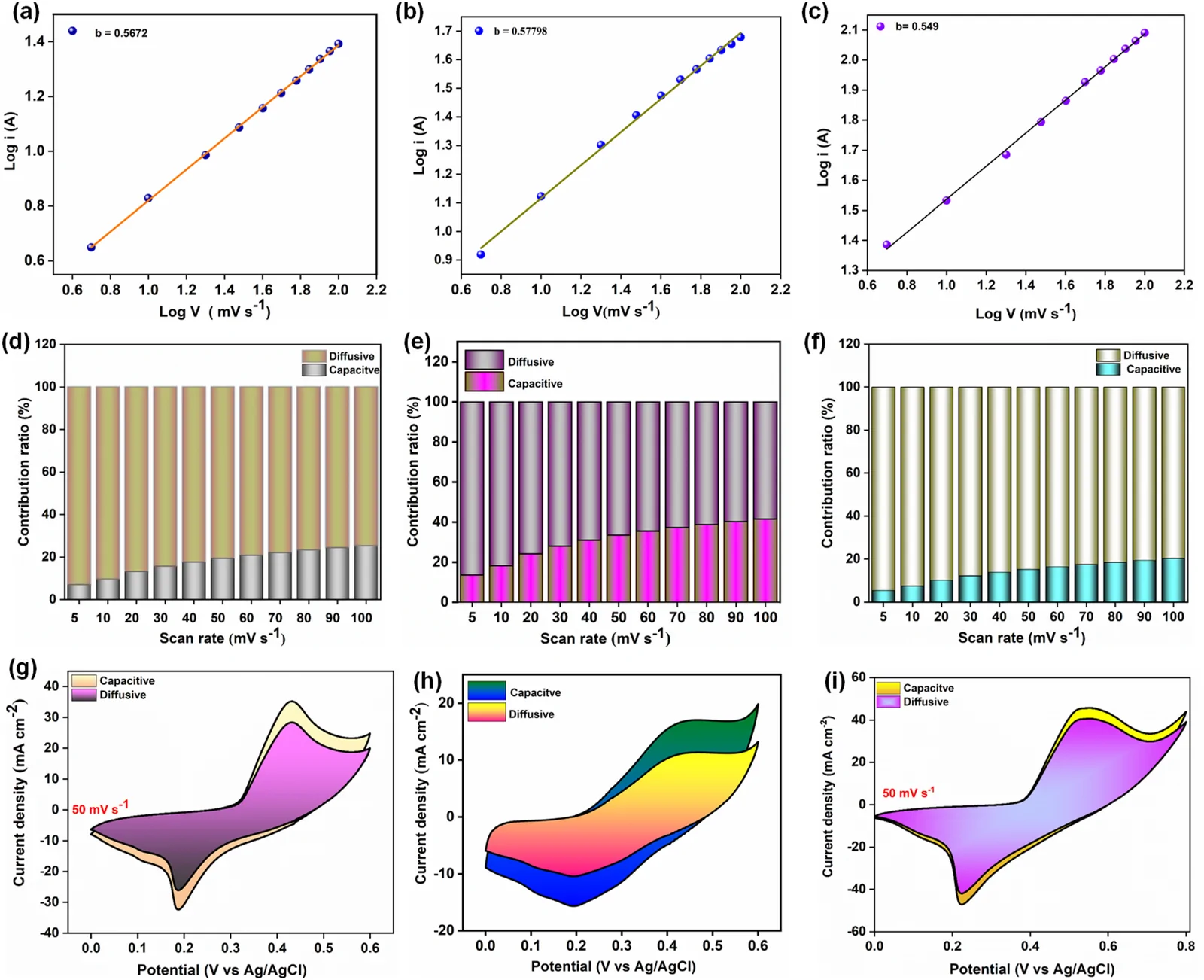
(a)–(c) *b*-Value (slope), (d)–(f) current contribution at 5–100 mV s^−1^, (g)–(i) capacitance and diffusion contribution at 50 mV s^−1^ of NiNd-MOF, MoS_2_, NiNd-MOF@MoS_2_.

The galvanostatic charge–discharge (GCD) measurements of Ni-MOF, Nd-MOF, MoS_2_, NiNd-MOF, and NiNd-MOF@MoS_2_ were performed at 1–7 A g^−1^. The GCD profiles exhibit non-linear charge–discharge behaviours with discernible potential plateaus, consistent with the faradaic features in the corresponding CV curves. At 1 A g^−1^, NiNd-MOF@MoS_2_ exhibits the longest discharge time among the investigated electrodes, consistent with its larger CV area and higher charge-storage capacity. The discharge time decreases with increasing current density, which can be associated with the reduced utilization of electroactive processes during the shorter charge–discharge timescale. *C*_s_ is calculated using the formula given below5
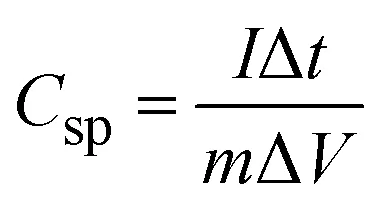


In this context, *I*, Δ*t*, *m*, and Δ*V* denote the current density, discharge time, active-material mass and voltage window, respectively. Ni-MOF exhibits *C*_sp_ of 576 F g^−1^ at 1 A g^−1^ arising from the reversible redox activity of electrochemically active Ni centers. Nd-MOF however, shows a relatively inert electrochemical utilization of active sites and poorer charge-storage kinetics, as indicated with a much lower *C*_sp_ of 102 F g^−1^ (Fig. S4(a) & (b)). Noticeably, the electrochemical performance can be enhanced with the introduction of Nd into the skeleton of Ni-MOF results in the *C*_sp_ of NiNd-MOF is 624 F g^−1^. The improvement is consistent with the CV results and it is due to the synergistic effect of Ni and Nd centers which increase the density of the electrochemically accessible active sites and positively influence the charge-storage process. The layered nanosheets MoS_2_ exhibits a high density of exposed surface sites and good accessibility of electrolyte, leading to the *C*_sp_ of 322 F g^−1^. More significantly, NiNd-MOF@MoS_2_ heterostructure showed the highest *C*_sp_ of 813 F g^−1^ which indicates the effectiveness of the bimetallic MOF frameworks together with the layered MoS_2_ nanosheets. The synergistic effects between components are responsible for the excellent electrochemical performance of NiNd MOF@MoS_2_ (Fig. 9(a)–(f)). Further to directly compare the electrochemical responses of NiNd-MOF, MoS_2_ and NiNd-MOF@MoS_2_ comparative CV measurements were performed at 100 mV s^−1^, while the corresponding GCD profiles were recorded at 1 A g^−1^ (Fig. S5). Among the three electrodes, NiNd-MOF@MoS_2_ exhibits the largest enclosed CV area and the longest discharge time. The *C*_sp_ at 1 A g^−1^ are 624, 322 and 813 F g^−1^ for NiNd-MOF, MoS_2_ and NiNd-MOF@MoS_2_, respectively. At the same current density, the *C*_sp_ of NiNd-MOF@MoS_2_ is 30.3% and 152.5% higher than that of NiNd-MOF and MoS_2_, respectively. These results demonstrate the enhanced charge-storage capability of the NiNd-MOF@MoS_2_ heterostructure relative to its individual components.

**Fig. 9:**
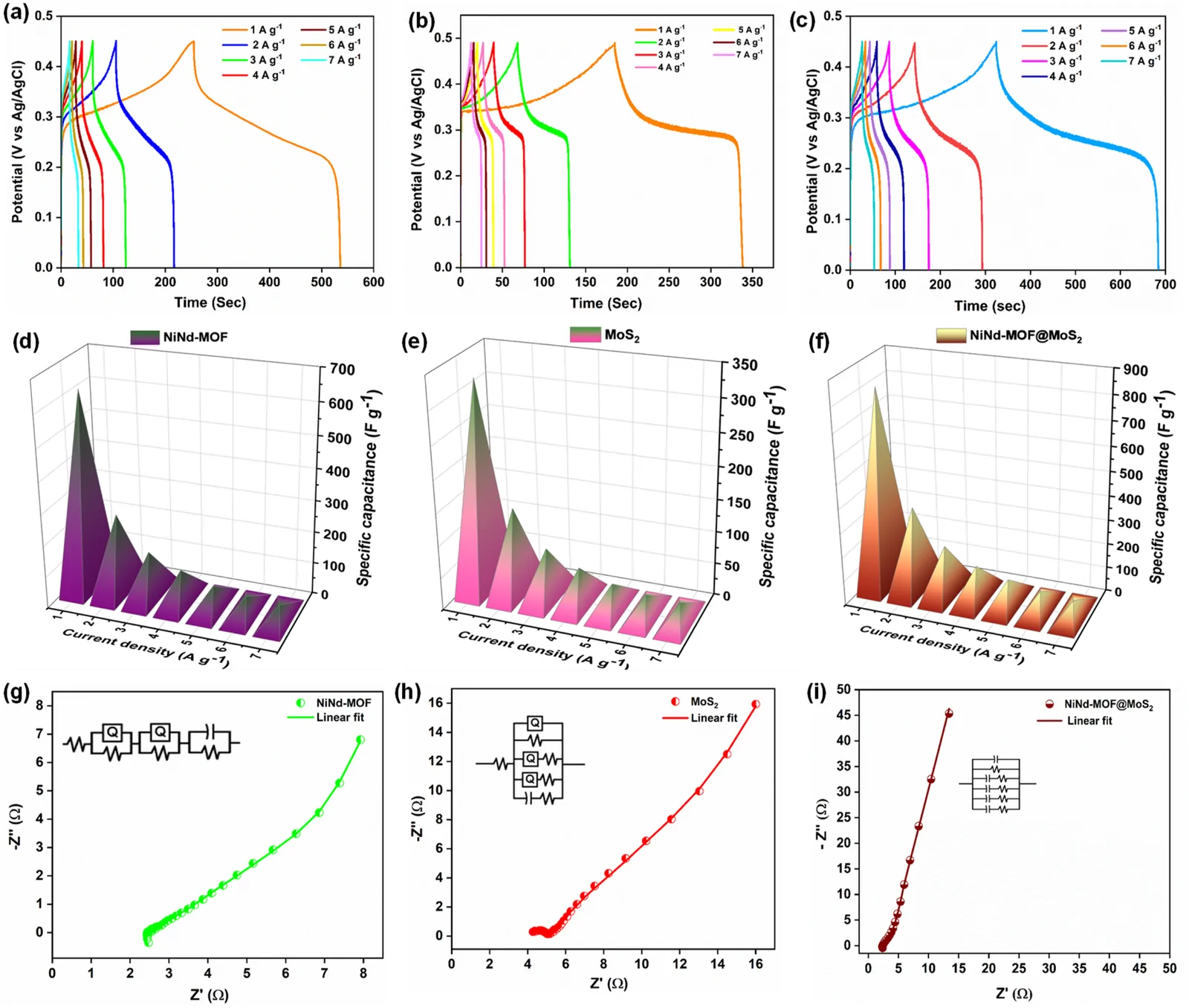
(a)–(c) GCD profiles, (d)–(f) *C*_sp_ at 1–7 A g^−1^, (g)–(i) Nyquist plots of NiNd-MOF, MoS_2_ and NiNd-MOF@MoS_2_.

Electrochemical impedance (EIS) of all the electrodes was studied by Nyquist plots from 100 kHz to 10 mHz. The high frequency arc is usually assigned to the interfacial resistance including the equivalent series resistance (*R*_s_). *R*_s_ values of Ni-MOF (3.9 Ω), Nd-MOF (4.9 Ω) Fig. S6(a) & (b), MoS_2_ (4.2 Ω), NiNd-MOF (2.6 Ω) and NiNd-MOF@MoS_2_ (2.3 Ω) (Fig. 9(g)–(i)). Among all electrodes, NiNd-MOF@MoS_2_ has the lowest *R*_s_ value with a steep low-frequency line, indicating good ion transfer as well as electrochemical reaction kinetics. Additionally, the Nyquist plots in the low frequency domain show inclined-to-steep responses, reflecting ion transport and non-ideal capacitive characteristics. The more vertical low frequency response of NiNd-MOF@MoS_2_ suggests weaker diffusion limitation and more capacitive response than the more inclined profiles. These characteristics are consistent with the presence of diffusion-related contributions to the low-frequency region impedance response.^74^ The EIS responses were further analysed and validated using the corresponding equivalent circuits insert within the graph. The cycling stability of the electrode for long term was examined up to 10 000 cycles. The capacitance retention of the electrode after cycling was 83.0%. Moreover, the coulombic efficiency was close to 100% in the subsequent cycles (Fig. S7(a) & (b)), which demonstrated good charge–discharge reversibility and electrochemical stability.

#### 3.3.2. Symmetric two-electrode system

The excellent electrochemical performance of NiNd-MOF@MoS_2_ in the three-electrode setup motivated its further evaluation in an assembled two-electrode configuration. The electrochemical behaviour of the assembled two-electrode configuration was studied by CV within 0–1.4 V with scan rates varying from 5–100 mV s^−1^ as presented in Fig. 10(a). The CV curves show quasi-rectangular profiles with apparent redox features, suggesting the coexistence of electrical double-layer capacitance (EDLC) and faradaic charge-storage contributions. Furthermore, the overall CV profiles remains well preserved with the increase of scan rate, indicating good electrochemical reversibility and rate capability. Fig. 10(b) depicts GCD curves at 1–4 A g^−1^ in the voltage window of 0–1.4 V. The 0–1.4 V operating window was selected based on the electrochemical behaviour observed for the individual electrode and subsequently evaluated in the assembled two-electrode configuration. The non-linear charge–discharge curves with well-defined voltage plateaus are in agreement with the redox features observed in the CV profiles. The assembled two-electrode system shows *C*_sp_ of 76.5 F g^−1^ at 1 A g^−1^. A distinct instantaneous voltage drop is observed at the charge–discharge transition, which is associated with the equivalent series resistance (ESR) of the assembled two-electrode system. The EIS gives an *R*_s_ of 4.5 Ω before cycling, which is an independent measurement of the series resistance of the assembled device.^75,76^

**Fig. 10:**
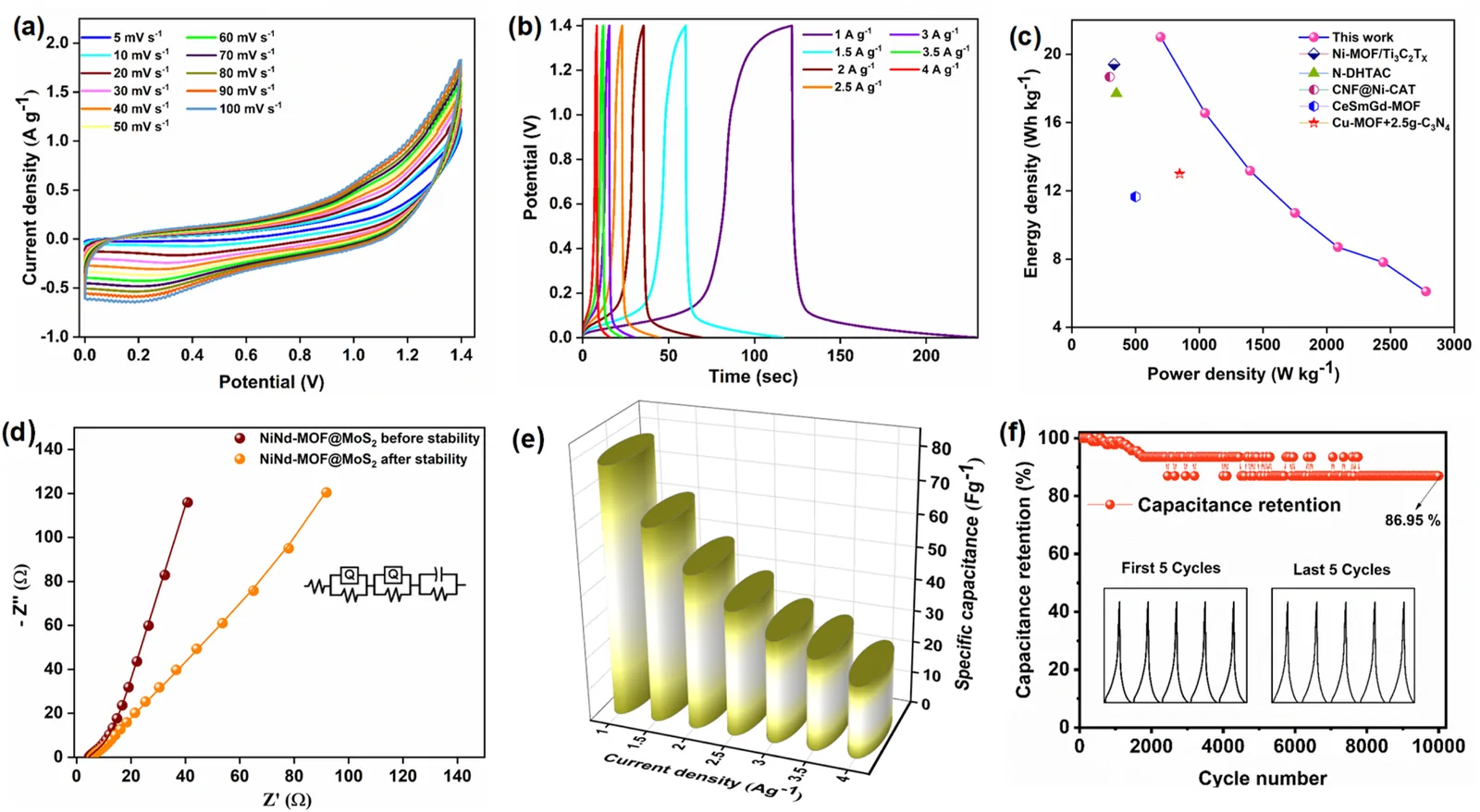
(a) CV curves at 5–100 mV s^−1^, (b) GCD profiles at 1–7 A g^−1^, (c) Ragone plot (d) EIS plot before and after stability (e) specific capacitance bar diagram representation, (f) capacitance retention for 10 000 cycles.

The *E*_d_ and *P*_d_ were evaluated from the GCD results using the following equations:6
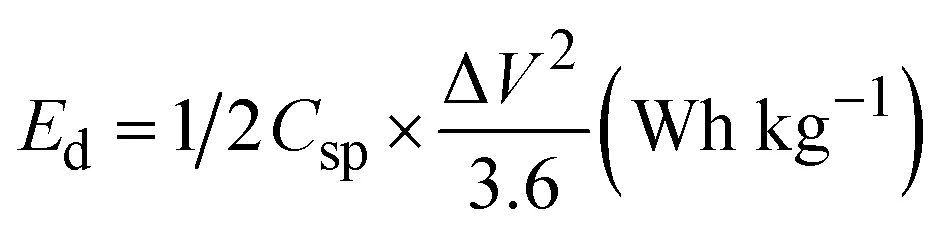

7 *P*_d_ = *E*_d_ × 3600/Δ*t* (W kg^−1^)

The *E*_d_ of 21 Wh kg^−1^ is achieved at the *P*_d_ of 696.7 W kg^−1^ in the Ragone plot for the assembled two-electrode setup. At a much higher *P*_d_ of 2779 W kg^−1^, *E*_d_ of 6.1 Wh kg^−1^ is retained, which is indicative of the system's ability to operate over a wide energy-power range. Additionally, relevant reported literature values (*E*_d_ & *P*_d_) have been added for comparison in Fig. 10(c).^77–81^Fig. 10(d) shows electrochemical impedance spectroscopy (EIS) measurement before and after cycling. The assembled two-electrode configuration has an (*R*_s_) of 4.5 Ω before cycling. Only a small increase of the resistance values is observed after the cycling test (*R*_s_) of 5.5 Ω which demonstrates that the assembled two-electrode configuration retains its electrochemical characteristics after long-term operation. The *C*_sp_ values at different current densities are summarized in Fig. 10(e). It is observed that the capacitance decreases gradually with increasing current density, which is generally ascribed to the decreased accessibility of electrochemically active sites at high charge–discharge rates. The assembled two-electrode configuration was subjected to long-term cycling stability for 10 000 charge–discharge cycles at 4.5 A g^−1^. The constructed two-electrode system exhibits a capacitance retention of 86.95% (Fig. 10(f)) of the initial capacitance with the coulombic efficiency remaining at ∼100% during the cycling process (Fig. S8). The results show the good cycling stability and reversibility of the NiNd-MOF@MoS_2_ assembled two-electrode configuration; added to that valuation with other MOF based electrode materials for SCs is concluded in Table 1.

**Table 1:** Assessment of stability performance of reported MOF-based SCs electrodes

Material	Electrolyte	*E*_d_ (Wh kg^−1^)	*P*_d_ (W kg^−1^)	Cycling stability (%)/cycles	Ref.
Ni-MOF/rGO	6 M KOH	0.82	20	90/3000	82
Mn_0.1_-Ni-MOF	6 M KOH	39.6	143.8	83.6/5000	83
NiCo MOF	3 M KOH	28.5	400.5	95.4/5000	84
rGO@Co/Mn MOF	2 M KOH	7.6	1515	72.1/10 000	85
**NiNd-MOF@MoS**_**2**_	**3M KOH**	**21**	**696.7**	**86.9/10 000**	**This work**

## 4. Device-level performance validation

A symmetric two-electrode supercapacitor (SSC) device was fabricated by using nickel foam (1 × 2 cm^2^) as the current collector with extended tabs for electrical connections. The fabricated electrode slurry was coated on two nickel-foam electrodes as the NiNd-MOF@MoS_2_ active material. Whatman filter paper was used as a separator and was saturated with 20 µL of 3 M KOH aqueous electrolyte. The symmetric device was fabricated in a face-to-face configuration with electrolyte-wetted separator sandwiched between two identically coated electrodes (Fig. 11). The practical use of the fabricated SSC is demonstrated by constructing a transistor assisted white light emitting diode (LED) switching circuit as shown in Fig. 12. In this arrangement, the SSC was first fed with a controlled voltage source. Then it acted as a temporary voltage source at the base terminal of a BC547 NPN transistor. The voltage stored in the SSC was used as the switching signal to control the on/off state of the transistor. The white LED was powered from a 7.2 V battery source. When the voltage across the base-emitter of the BC547 transistor reached a certain point, it turned on and current from the battery flowed through the white LED lighting it up. The continuous LED illumination at 60, 120, 180, 240 and 300 s shows that the fabricated SSC has enough charge to operate an electronic switching circuit for a long period. Thereby demonstrating the possibility of using the fabricated SSC in low power switching and energy-storage assisted electronic devices.

**Fig. 11:**
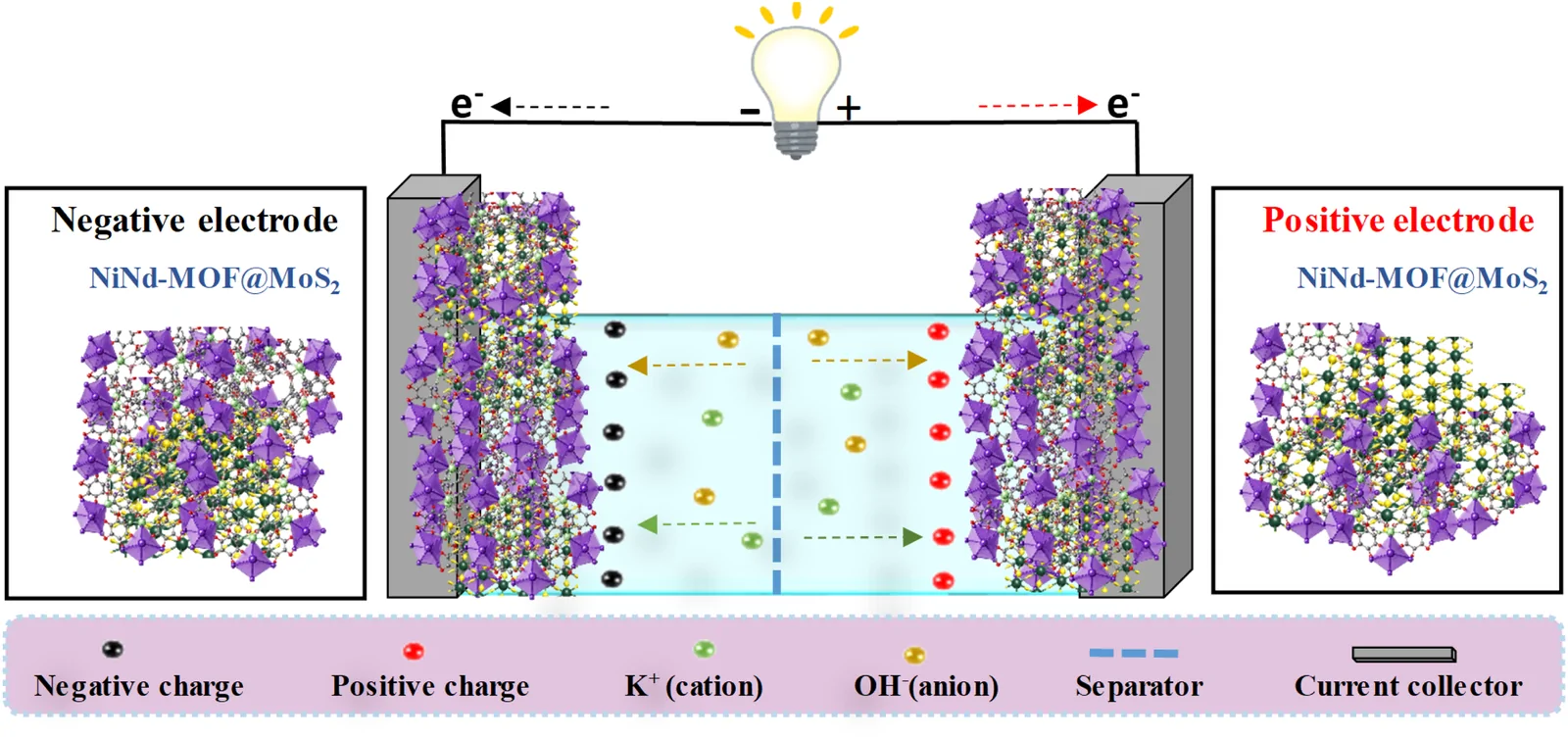
Schematic illustration of the NiNd-MOF@MoS_2_ SSC including the electrode architecture, electrolyte ion migration, charge storage and external electric connection.

**Fig. 12:**
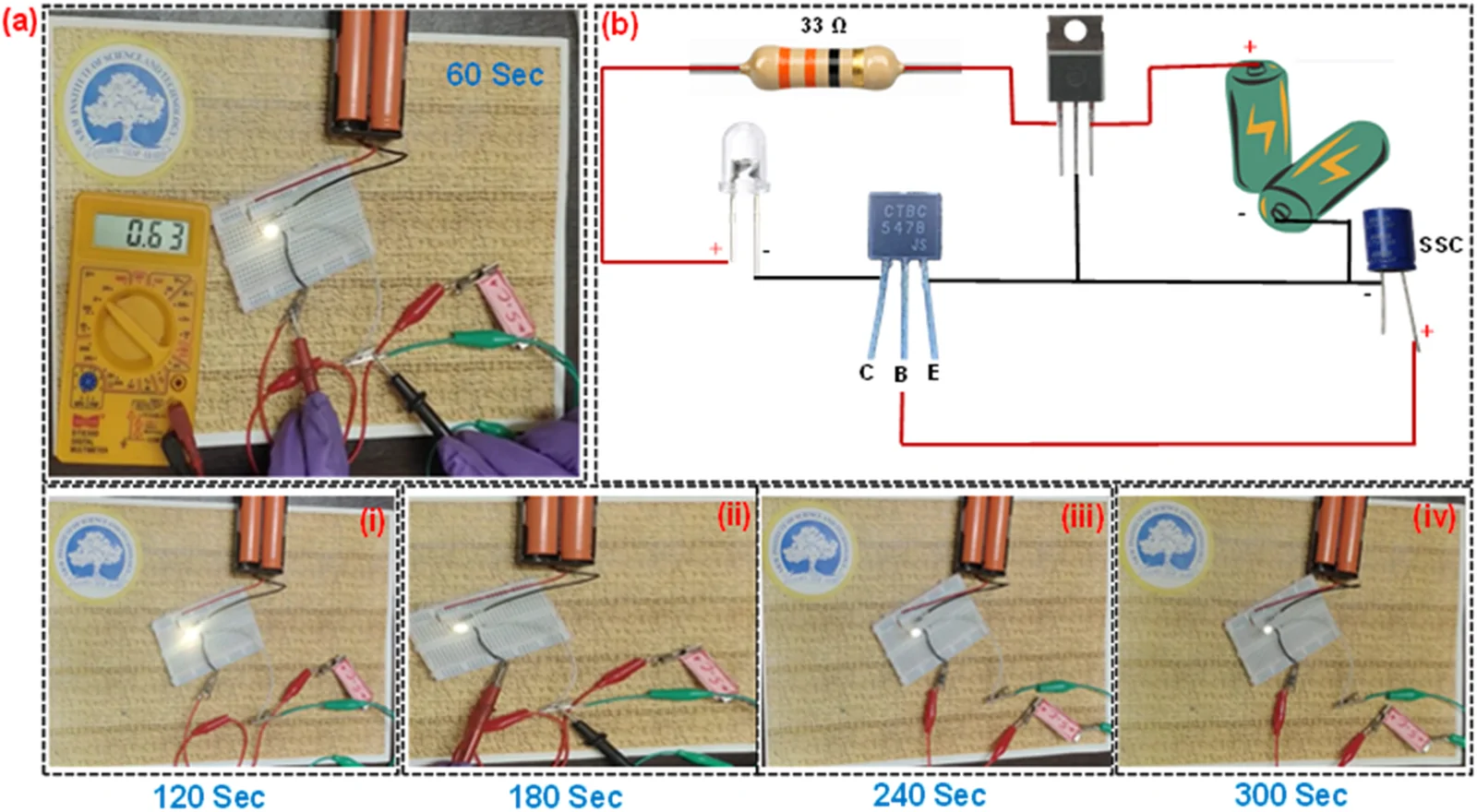
(a) Photograph of the transistor-assisted white LED demonstration with the fabricated SSC. (b) Schematic diagram of the experimental circuit. (i)–(iv) Successive photographs taken with the white LED continuously illuminated after 120, 180, 240, and 300 s, respectively, demonstrating the charge-retention capability of the fabricated SSC in a transistor switching circuit.

## 5. Conclusions

The resultant NiNd-MOF@MoS_2_ study introduces a novel hetero-structure that presents a rationally engineered architecture in which the bimetallic NiNd-MOF is intimately integrated with MoS_2_ nanosheets. The increased exposure of accessible active sites facilitates rapid electron transport and efficient ion diffusion, thereby enhancing the overall electrochemical performance beyond that of the individual components. Thus, a significantly upgraded *C*_s_ of 813 F g^−1^, is achieved for NiNd-MOF@MoS_2_, in contrast to 624 F g^−1^, achieved in NiNd-MOF. By taking advantage of these attributes, the SSC made-up with NiNd-MOF@MoS_2_ yields a *C*_s_ of 76.5 F g^−1^ at 1 A g^−1^, coupled with a remarkable *E*_d_ of 21 Wh kg^−1^ and *P*_d_ of 696.7 W kg^−1^ with excellent cycling robustness displaying 86.95% capacitance retention and 100% coulombic efficiency over 10 000 cycles. Practical applicability of the material is confirmed by a transistor-driven LED circuit, which displays steady voltage retention and consistent charge–discharge behaviour, indicating reliable energy delivery for low-power electronic devices. In conclusion, overall results demonstrate that the NiNd-MOF@MoS_2_ heterostructure significantly outperforms its individual components and epitomizes a promising electrode material for high-performance SSC.

## Conflicts of interest

The authors declare that they have no known competing financial interests or personal relationships that could have appeared to influence the work reported in this paper.

## Supplementary Material

RA-016-D6RA07975J-s001

## Data Availability

Data will be made available on request. Supplementary information (SI): details of the materials, synthesis procedures, electrode preparation, electrochemical measurements, and characterization techniques, along with additional elemental composition data and supplementary electrochemical results, including cyclic voltammetry, *b*-value analysis, capacitive and diffusion-controlled contributions, galvanostatic charge–discharge profiles, specific capacitance, electrochemical impedance spectra, and cycling stability and coulombic efficiency data. See DOI: https://doi.org/10.1039/d6ra07975j.
