# Supplementary material for: Interfacial engineering of an amino-functionalized bimetallic NiNd-MOF decorated MoS2 heterostructure for enhanced symmetric supercapacitor performance
Source: RSC Adv. 2026 Oct 15;16(47):63120–34. doi: 10.1039/d6ra07975j (PMC13670516; doi:10.1039/d6ra07975j)
Supplement: Supplementary file 1 [file RA-016-D6RA07975J-s001.pdf]

Supplementary Information (SI) for RSC Advances.
This journal is © The Royal Society of Chemistry

Interfacial Engineering of an Amino-Functionalized Bimetallic NiNd-MOF decorated MoS_2 Heterostructure for Enhanced Symmetric Supercapacitor Performance

Oviya Annamalai[a], Bhavani Sankaran[a], T. Kalaivani[a]*

[a] Department of Physics and Nanotechnology, College of Engineering and Technology, SRM Institute of Science and Technology, Kattankulathur 603203, Chengalpattu District, Tamil Nadu, India.

*Corresponding author Mail ID: kalaivat@srmist.edu.in

SUPPORTING INFORMATION

1. Materials

AR grade of $Ni(NO_3)_2.6H_2O$ (purity 99%), $Nd(NO_3)_3.6H_2O$ (purity 99%), 2- Aminoterephthalic acid (purity 99%), Dimethylformamide $(CH_3)_2NC(O)H$, Ethanol (C_2H_6O), Ammonium molybdate tetrahydrate $(NH_4)_6Mo_7O_{24}.4H_2O$ (purity 99%), Thiourea (CH_4N_2S), KOH and NMP were obtained from SRL. Nickel foam was sourced from Vritra Technologies, while Carbon black and PVDF were from Alfa Aesar.

1.1. Synthesis of NiNd-MOF

Initially 50 mL equal volumes (1:1) of ethanol and N, N-dimethylformamide (DMF) was stirred together and 1.458 g of 2-Aminoterephthalic acid (NH_2-BDC) was added, and then stirred for 30 minutes. Additionally, 0.9845 g of $Ni(NO_3)_2{\cdot}6H_2O$ and 0.968 g of Nd $(NO_3)_3{\cdot}6H_2O$ were introduced, and the mixture was under continuous agitation until it was well mixed. Then it was transferred to autoclave and heated at 140 °C-24 hr, and cooled naturally to room temperature. After that the brownish residue was thoroughly washed using DI and ethanol at several intervals and dried for 12 hr at 80 °C to obtain NiNd-MOF and the synthesis process is depicted in **Fig. 1**.

1.2. Synthesis of MoS_2

A simple hydrothermal method was used to synthesize MoS_2. Initially, 0.856 g Ammonium molybdate tetrahydrate and 5.127 g Thiourea were dissolved in 80 mL DI and magnetically stirred for 1 hr to get a homogeneous precursor solution. The resulting precursor solution was transferred into a Teflon-lined stainless steel autoclave and kept at 180 °C for 24 hr under hydrothermal

conditions and then the autoclave was cooled down to room temperature naturally. The precipitate was separated by centrifugation, washed several times with DI and ethanol to remove residual impurities and dried at 70 °C for 12 hr to give the final product.[1] The resultant MoS_2 nanosheet is outlined in **Fig. 1**.

2. Electrode preparation and electrochemical measurements

Electro active material was blended by vigorous mixing with carbon black as an electronic conducting agent and Polyvinylidene fluoride (PVDF) as binder. The mixture, 8:1:1 mass percentage of the NiNd-MOF@MoS_2, PVDF, and carbon black, respectively, served as the substrate for electrode assembly and associated electrochemical studies. The powdered blend was thoroughly mixed with 100 μL of NMP to form a highly concentrated viscous suspension. Then the composite paste was drop-casted onto 0.5 cm x 1 cm nickel foam substrate softly for all the electrochemical experiments and served as the working electrode[2]. NiNd-MOF@MoS_2 was coated on two Ni-foam electrodes with geometric area of 1 cm × 1 cm for the symmetric two-electrode setup. The loading of active-material was ~2 mg for each electrode, which corresponds to a 1:1 mass ratio and a total mass of active-material of ~4 mg. 3 M KOH was used as the electrolyte. For device level electrochemical measurements, the two electrodes were positioned facing each other. The current density was calculated on the basis of total active-material mass of ~ 4. All the electrode performance assessment were executed using a Biologic SP-300 electrochemical workstation in an common three and two-electrode setup, platinum wire as counter electrode, while an Ag/AgCl electrode as the reference electrode. This comprehensive evaluation, including cyclic voltammetry (CV), galvanostatic charge-discharge (GCD), electrochemical impedance spectroscopy (EIS), cyclic stability tests enables rapid and accurate analysis of the NiNd-MOF@MoS_2 for charge storing evaluation.

3. Characterisation techniques

The crystal structures were studied by PANalytical diffractometer XPert Pro with Cu Kα radiation (λ = 0.1540 nm). The functional groups and bond interactions were characterized by Fourier Transform Infrared (FTIR) spectroscopy (FTIR Spectrometer-SHIMADZU, IRTRACER 100).

Surface morphologies and microstructures were investigated by high resolution scanning electron microscopy (HR-SEM; Thermoscientific Apreo S) and high resolution transmission electron microscopy (HR-TEM; JEOL Japan, JEM-2100 Plus). The elemental composition and oxidation states of the nanocomposites were investigated by X-ray photoelectron spectroscopy (XPS; PHI Versaprobe III). Nitrogen adsorption and desorption analysis was performed using an Autosorb iQ-Chemisorption (and Physisorption) Gas Sorption Analyzer. The surface area was analyzed by the Brunauer-Emmett-Teller (BET) method and the pore size distribution by the adsorption-desorption isotherm.

4. Results and discussion

4.1 Morphological analysis

Element	Atomic number	Net counts	Mass concentration (%)	Norm. mass concentration (%)	Norm. atomic concentration (%)
**C**	6	1695	27.41	29.3	50.73
**N**	7	111	1.94	2.08	3.08
**O**	8	3249	21.17	22.7	29.41
**S**	16	16349	14.96	16.0	10.37
**Ni**	28	637	2.73	2.93	1.03
**Nd**	42	12961	19.56	20.97	4.53
**Mo**	60	1952	5.51	5.90	0.85
		Sum	93.28	100.00	100.00

Fig.S1. Elemental composition in terms of wt % and at %.

5. Electrochemical Analysis

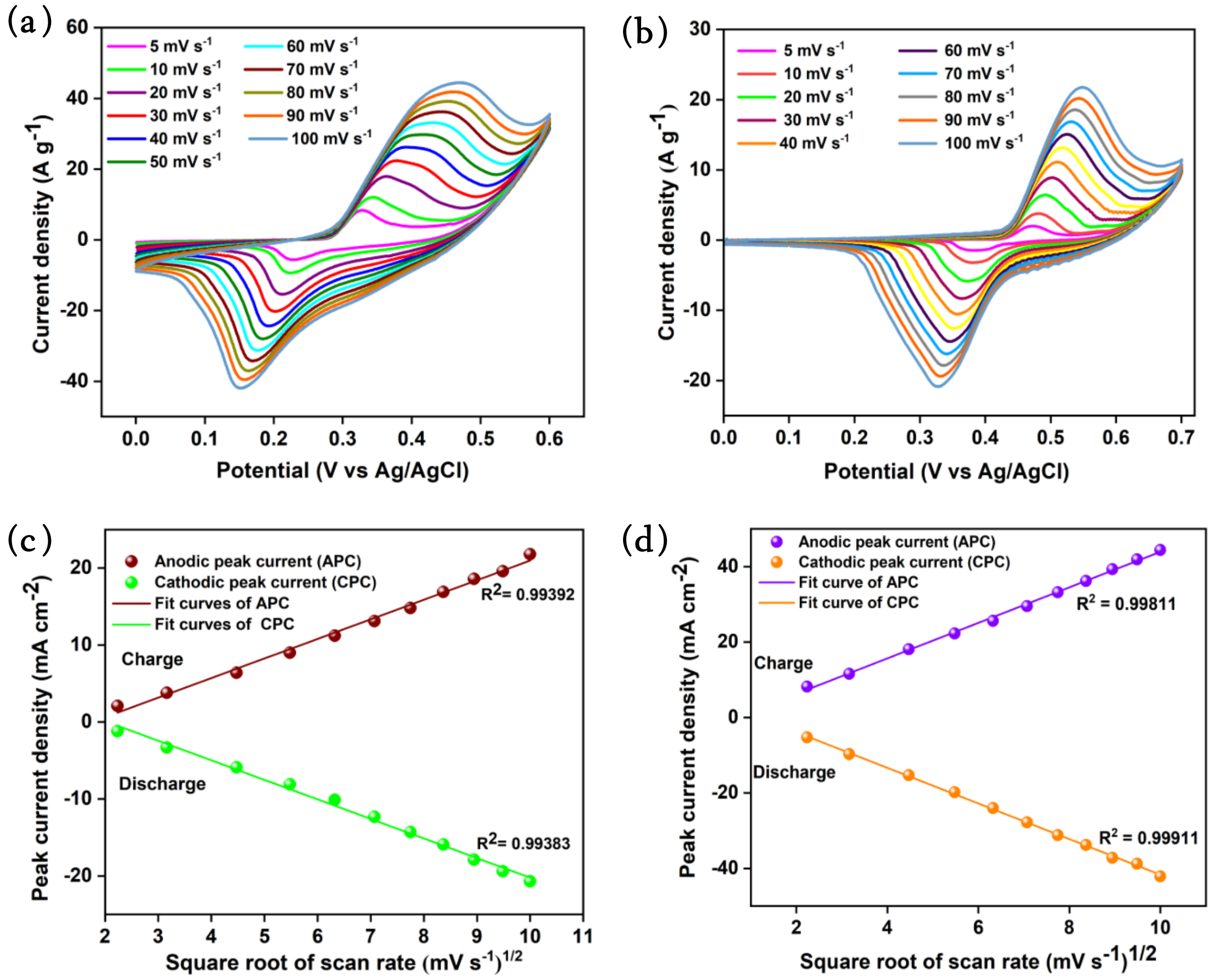

Fig.S2. (a&b) CV of Ni-MOF, Nd-MOF, (c&d) i_p vs $v^{1/2}$

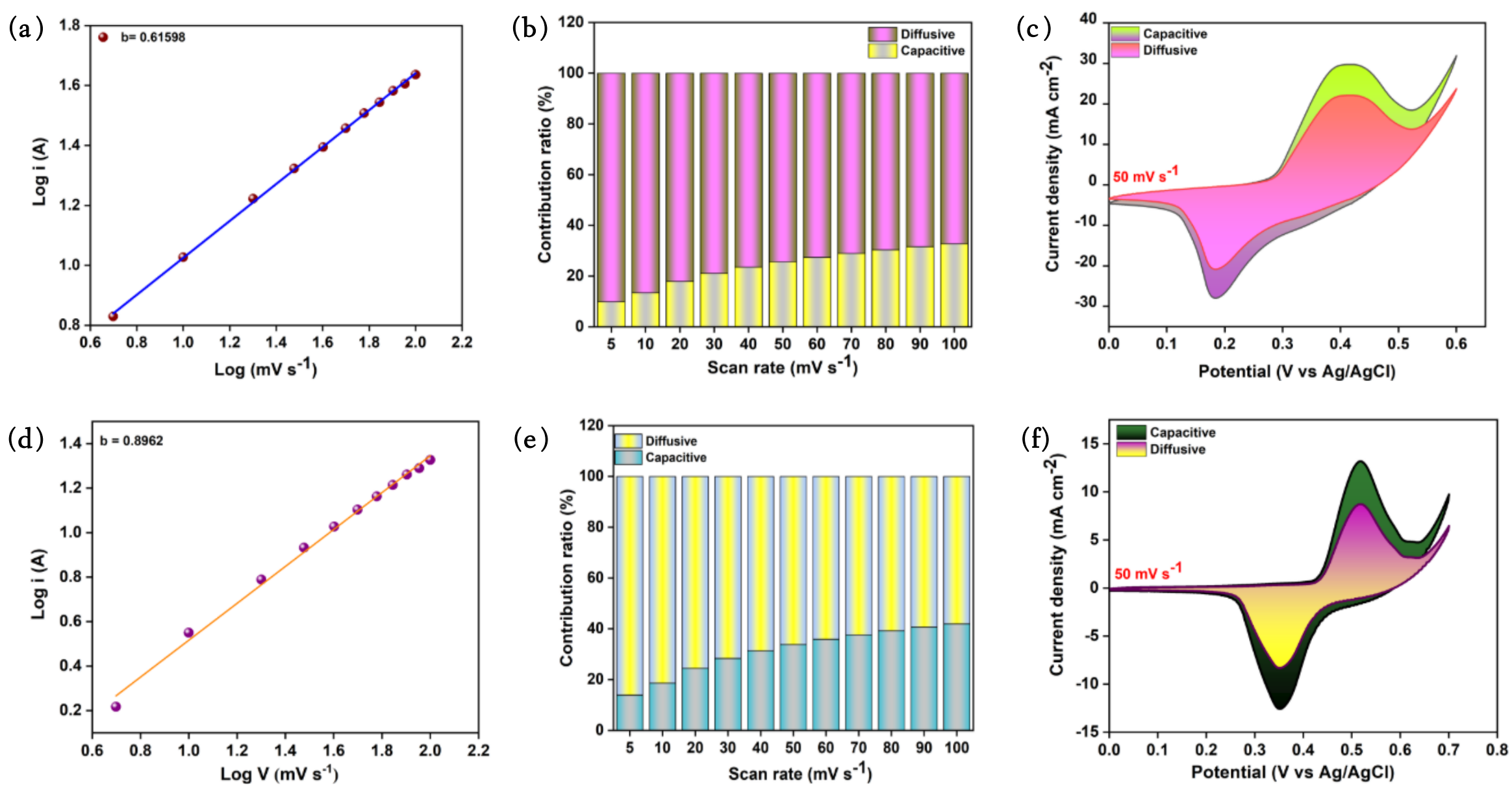

Fig.S3. (a-c) b-value (slope), current contribution at 5-100 mV s^{-1}, capacitance and diffusion contribution at 50mV s^{-1} of Ni-MOF, (d-f) b-value (slope), current contribution at 5-100 mV s^{-1}, capacitance and diffusion contribution at 50mV s^{-1} of Nd-MOF.

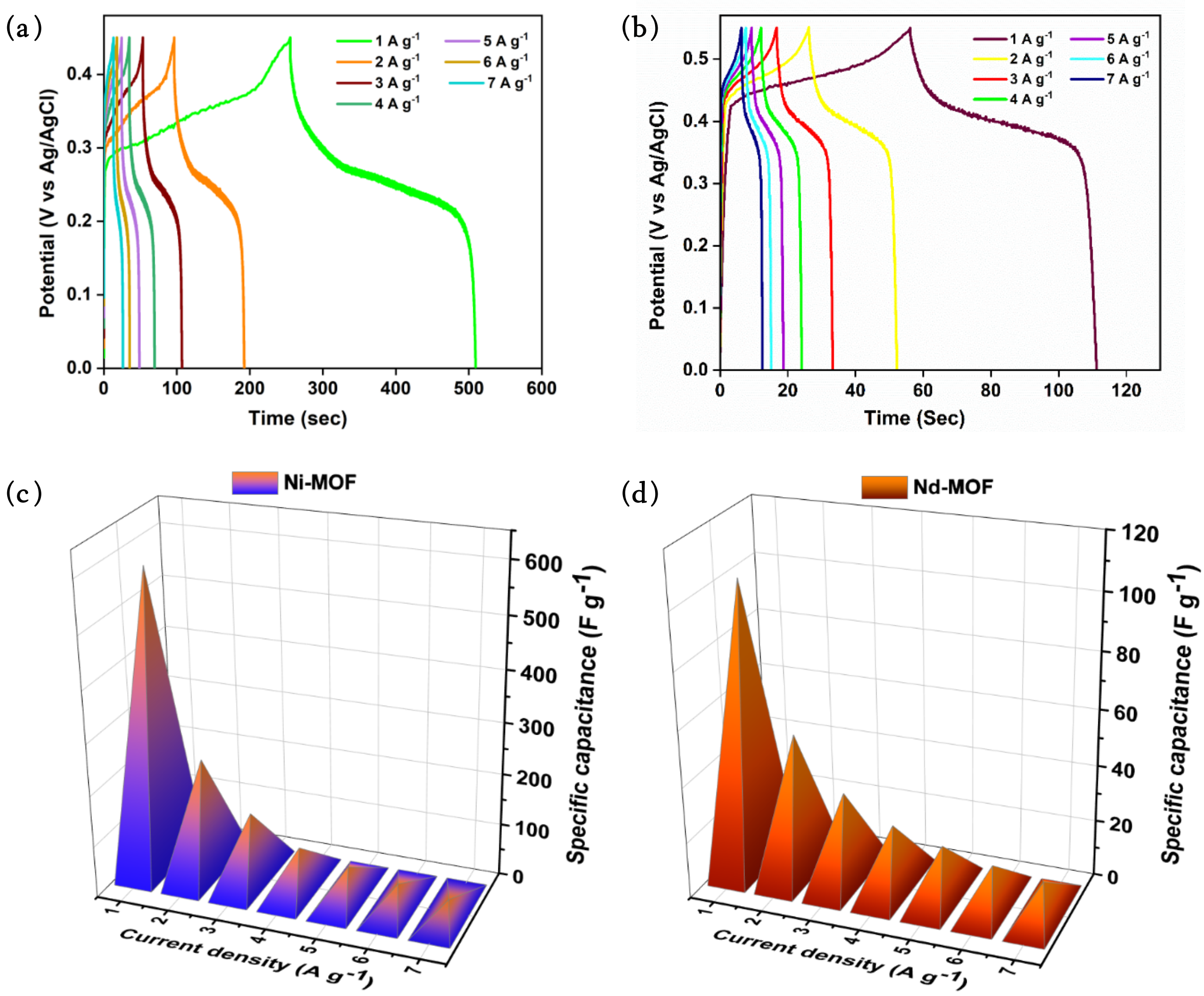

Fig.S4. (a&b) GCD curves at different current densities and, (c&d) specific capacitance at various current densities of Ni-MOF and Nd-MOF respectively.

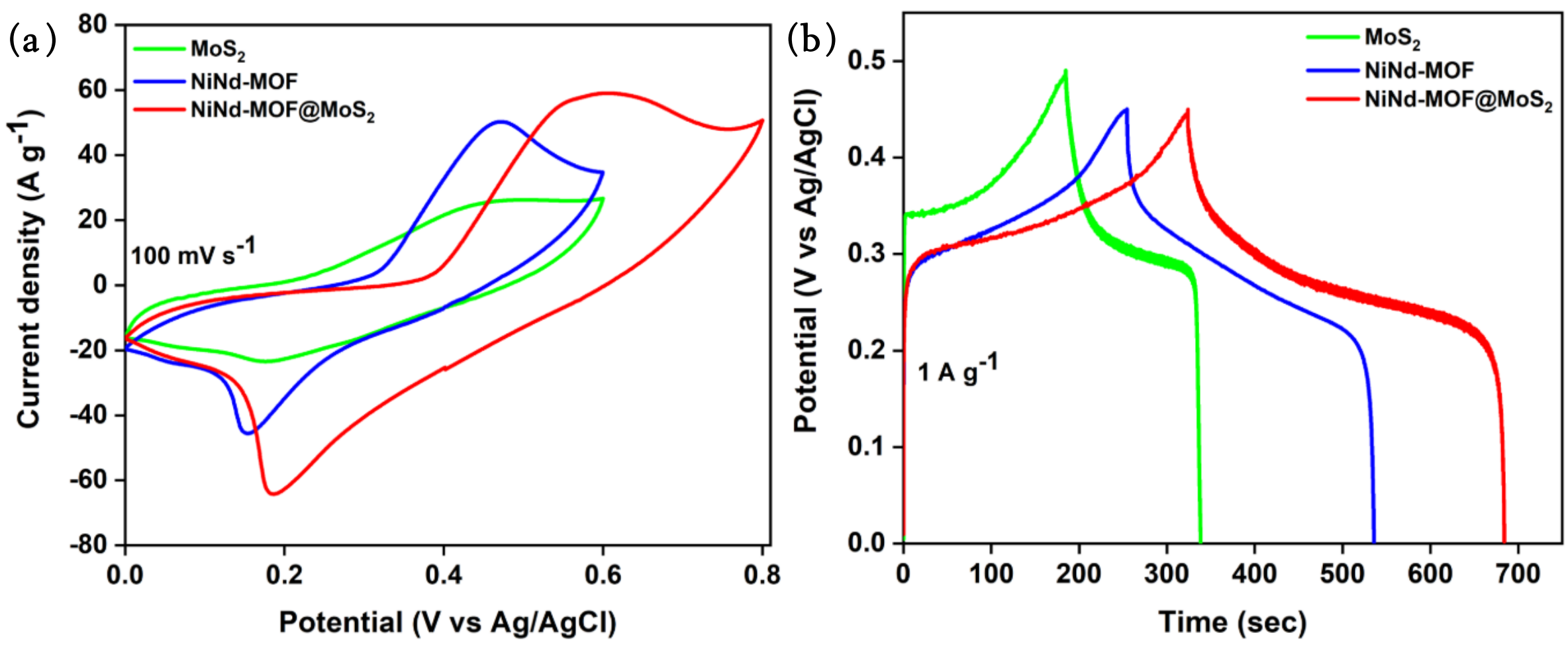

Fig.S5. (a) Comparative CV profiles at 100 mV s^{-1}, (b) Comparative GCD profiles at 1 A g^{-1} of MoS_2, NiNd-MOF &NiNd-MOF@MoS_2

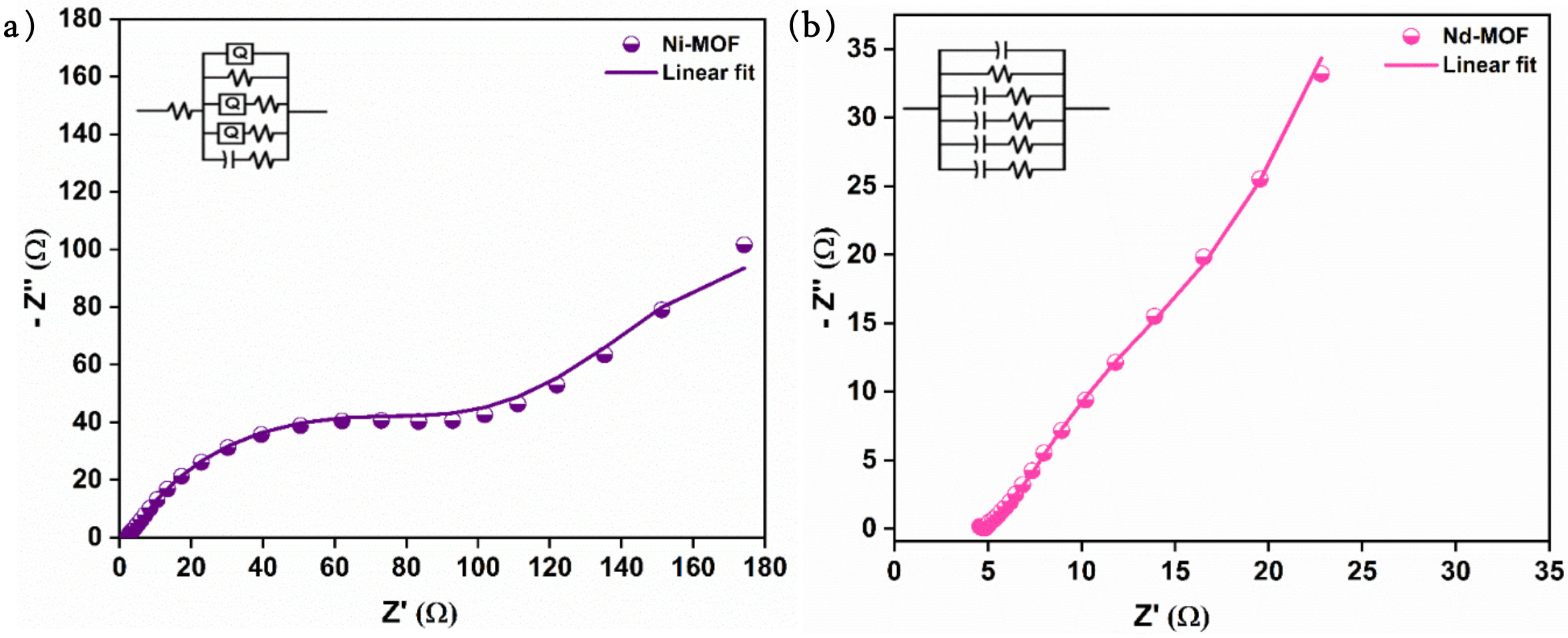

Fig. S6. (a) Nyquist plots of Ni-MOF, (b) Nd-MOF.

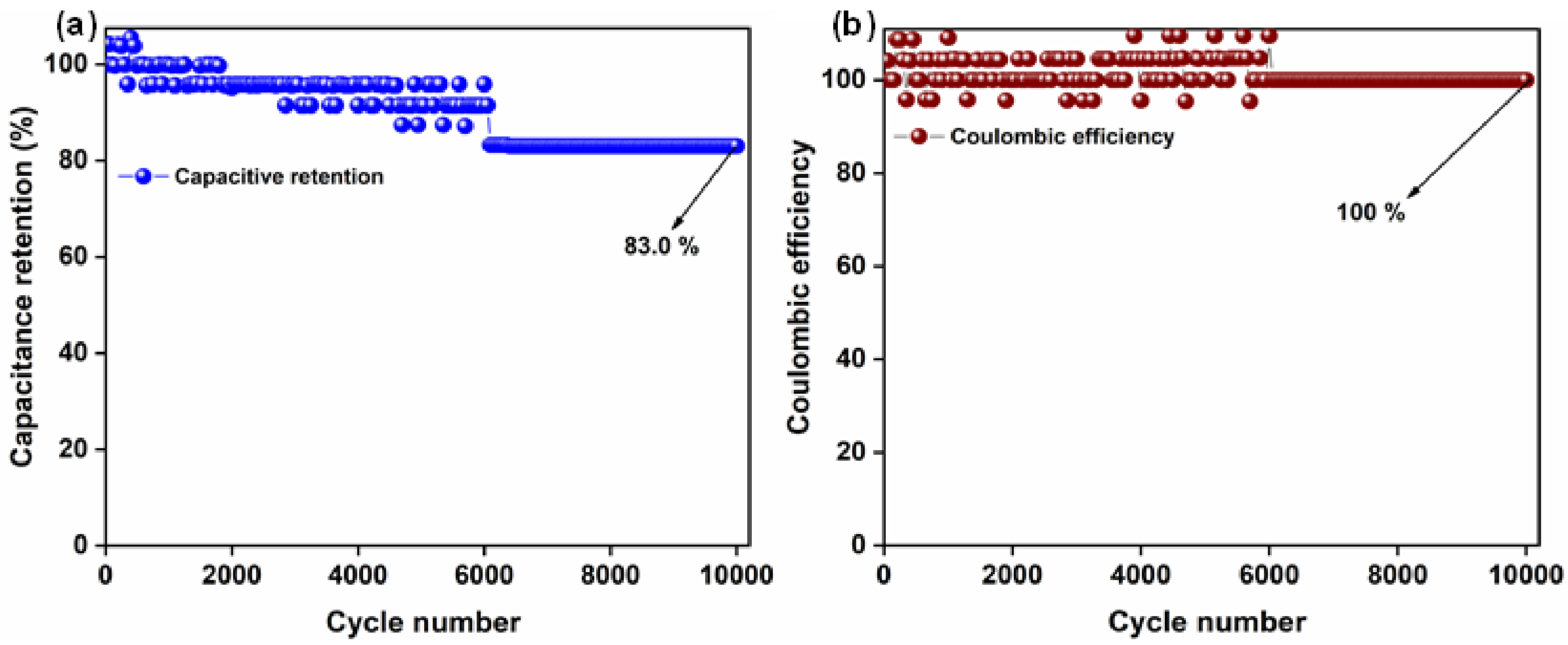

Fig. S7. (a) Cycling stability, (b) coulombic efficiency for 10000 cycles for three electrode system.

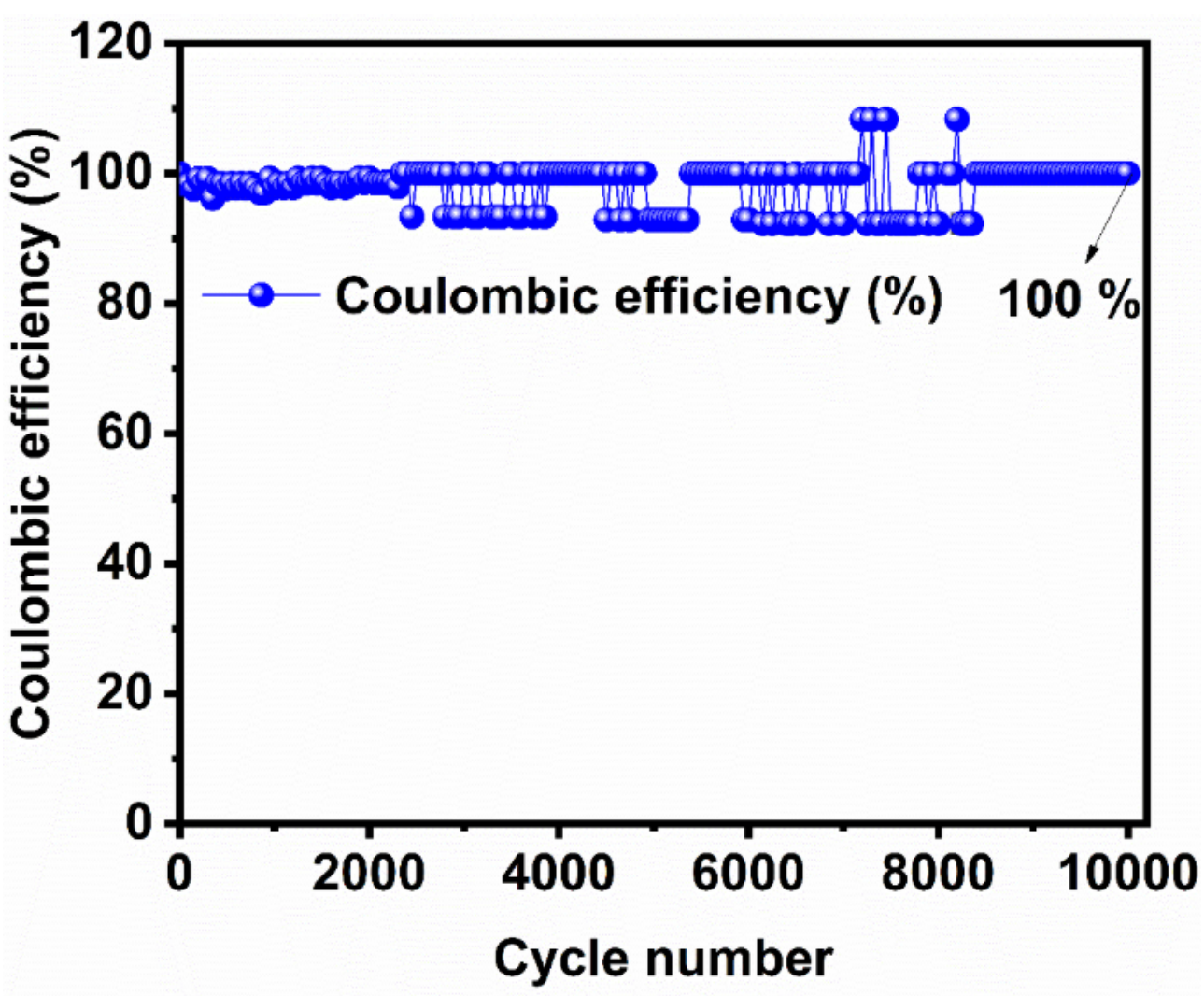

Fig. S8. Coulombic efficiency for 10,000 cycles for symmetric two electrode system.

References

1. S. Chinnasamy, J. Madhavan, P. Karthikesan and A. Mani, *J. Power Sources*, 2025, **642**, 236924.
2. A. Kaliyaraj Selva Kumar, Y. Zhang, D. Li and R. G. Compton, *Electrochem. Commun.*, 2020, **121**, 106867.
